# What level of automation is “good enough”? A benchmark of large language models for meta-analysis data extraction

**DOI:** 10.1017/rsm.2025.10066

**Published:** 2026-01-26

**Authors:** Lingbo Li, Anuradha Mathrani, Teo Susnjak

**Affiliations:** School of Mathematical and Computational Sciences, Massey University, New Zealand

**Keywords:** automated meta-analysis, data extraction, evidence synthesis, human-in-the-loop, large language models (LLMs), prompt engineering

## Abstract

Automating data extraction from full-text randomized controlled trials for meta-analysis remains a significant challenge. This study evaluates the practical performance of three large language models (LLMs) (Gemini-2.0-flash, Grok-3, and GPT-4o-mini) across tasks involving statistical results, risk-of-bias assessments, and study-level characteristics in three medical domains: hypertension, diabetes, and orthopaedics. We tested four distinct prompting strategies (basic prompting, self-reflective prompting, model ensemble, and customized prompts) to determine how to improve extraction quality. All models demonstrate high precision but consistently suffer from poor recall by omitting key information. We found that customized prompts were the most effective, boosting recall by up to 15%. Based on this analysis, we propose a three-tiered set of guidelines for using LLMs in data extraction, matching data types to appropriate levels of automation based on task complexity and risk. Our study offers practical advice for automating data extraction in real-world meta-analyses, balancing LLM efficiency with expert oversight through targeted, task-specific automation.

## Highlights

### What is already known?

- Extracting data manually from randomized controlled trials (RCTs) for meta-analyses is known to be a time-consuming and error-prone part of evidence synthesis.
- Previous studies have investigated automation for data extraction using machine learning and natural language processing, but these methods often faced challenges with varied reporting formats that needed extensive domain-specific adjustments.
- Large language models (LLMs) have recently shown potential in information extraction, but their role in complex data extraction specifically for meta-analysis has not been widely studied.

### What is new?

- This study presents the first comprehensive evaluation of LLMs for full-text RCTs’ data extraction across multiple clinical domains, information types, and task structures in meta-analysis through three state-of-the-art LLMs (Gemini-2.0-flash, Grok-3, and GPT-4o-mini).
- We show that customized, task-specific prompts and model ensembles provide distinct but complementary benefits for automated data extraction. Customized prompts help models capture more complete information, particularly for statistical results, resulting in substantially improved coverage (up to 15% more relevant data retrieved). Ensemble methods enhanced output diversity, allowing weaker models to compensate for missing content.
- Based on observed variation in extraction difficulty and risk, we propose a practical three-tier classification of data types by their suitability for automation. This framework guides when to automate and when to involve human oversight to offer actionable insights for integrating LLMs into meta-analytic workflows.

### Potential impact for RSM readers

- LLMs can meaningfully assist in data extraction workflows by rapidly generating outputs, but they are not yet reliable enough for full automation, especially in tasks requiring high completeness or detailed interpretation. Their role, at least for now, is to assist rather than replace human reviewers.
- For researchers, integrating LLMs in data extraction can help reduce workload and accelerate review timelines. However, critical tasks, especially those involving complex statistical reporting, still require human oversight to ensure accuracy and completeness.
- For developers, our findings highlight the urgent need to be mindful of weakness in current LLM-based systems. Future development should focus on incorporating task-specific prompts and hybrid approaches that combine LLMs with structured extraction tools to improve reliability and coverage.

## 1 Introduction

Meta-analysis is a gold standard in evidence-based medicine, combining quantitative findings from clinical trials to guide healthcare decisions.^1^ ^,^ ^2^ Accurate data extraction is a foundational component in meta-analyses, as it directly affects the quality of findings and validity of the conclusions. Traditionally, this step has been highly manual and resource-intensive,^3^ requiring multiple reviewers to identify and double-check key details from each study.^4^ Errors at this stage are common and can compromize the accuracy of meta-analytic conclusions.^5^ Furthermore, with the growing volume of the published research, the need for efficient, accurate, and reliable automation methods capable of scalability has become more critical.^6^

Over the years, researchers have explored a range of automated data extraction approaches to alleviate the manual burden. Early efforts used rule-based systems with manually designed patterns to extract trial elements, such as sample sizes and interventions.^7^ ^,^ ^8^ While these showed that automated extraction was possible, they were constrained by the variability of reports, making them challenging to maintain or expand. To overcome such drawbacks, classical machine learning and specialized natural language processing techniques,^7^ ^,^ ^9^ ^–^ ^12^ such as named entity recognition and supervised classification to extract population, intervention, comparator, and outcome (PICO) elements, are being employed. For example, BERT-based models, like those described by Mutinda et al.,^13^ offered better adaptability compared to rule-based systems. However, these earlier approaches needed annotated datasets,^7^ ^,^ ^9^ ^,^ ^12^ were often designed for narrow or specific objectives,^9^ ^,^ ^10^ and struggled with complex full-text documents.^7^ ^,^ ^11^ ^,^ ^12^ A recent literature review has noted that 84% of extraction methods focused on abstracts, with only 25% attempting to process full-text trial reports.^14^ Furthermore, only a small proportion of projects produced widely accessible tools (approximately 8% of methods had publicly available implementations),^14^ highlighting the difficulty of creating broadly applicable solutions. Despite these challenges, semi-automated tools like RobotReviewer^15^ and AutoLit (https://about.nested-knowledge.com/) have emerged. RobotReviewer was among the first systems to address full-text data extraction and risk-of-bias assessment simultaneously. Though it aids reviewers by summarizing participant details and methodological quality, it does not fully capture numerical outcomes essential for meta-analysis. AutoLit incorporates AI into the entire systematic review process, providing tools, such as inclusion prediction and NLP-assisted data extraction. The platform’s value lies in its ability to streamline workflows, ensuring that extracted data flow directly into meta-analysis and visualization components (the “Synthesis” module of the platform). Although AutoLit streamlines many aspects of the systematic review process, its outputs still require substantial human oversight.^16^ This is evident when extracting complex or numerical data from tables.^17^

More recently, large language models (LLMs) powered systems offer greater adaptability. Tools like MetaMate^18^ employ LLMs in a structured extraction pipeline with verification steps, achieving strong accuracy for participant and intervention data. In controlled settings, MetaMate attained F1 scores similar to those of human coders, including accurately parsing numerical expressions. However, it has not yet addressed full outcome extraction and has primarily been evaluated in the educational domain. The advent of general LLMs (such as ChatGPT,^19^ Claude,^20^ and others) has introduced powerful new ways to automate data extraction. With LLMs’ ability to understand context and perform tasks without extensive training from scientific texts, researchers are currently evaluating LLMs both as independent extractors and within human-in-the-loop workflows.^18^ ^,^ ^21^ ^–^ ^23^ Studies show promising results: GPT-4 achieved 82% accuracy extracting trial characteristics in biomedical randomized controlled trials (RCTs),^24^ and Claude successfully extracted binary outcome data with around 70%–75% accuracy.^23^ Konet et al. showed Claude 2 can achieve 96.2% data elements correctness in ten PDF articles.^22^ However, their application to structured data extraction for meta-analysis has yet to be widely tested at the scale and complexity required for real-world use involving heterogeneous document types, especially when extracting detailed statistical information needed for meta-analyses.

This study investigates the extent to which current LLMs can reliably extract the structured data from raw scientific papers required for automated meta-analysis (AMA), and further examines how extraction demands differ across statistical results, risk-of-bias assessments, and study-level categories of data, as well as how these data types impact extraction performance. We sought answers to these goals across three heterogeneous medical fields, namely, hypertension, diabetes, and orthopaedics, maximizing the generalizability of our research. In our work, we evaluated the data extraction performance of three advanced models (Gemini-2.0-Flash, Grok-3, and GPT-4o-mini) against a human-annotated ground-truth data, while exploring a variety of prompting and model-output aggregation strategies. Given the research momentum toward automating all parts of the meta-analysis process, our findings shed important light on the performance profile and the existing capabilities and limitations of current LLMs in real-world data extraction tasks for evidence synthesis purposes. Therefore, the contributions of this study are as follows:

- Comprehensive feasibility assessment: We provide the first in-depth benchmark of LLMs for full-text data extraction in meta-analysis across multiple clinical domains, information types, and task structures.
- Modular performance optimization: We demonstrate that prompt specialization and model output aggregation can yield distinct and complementary gains, establishing a modular strategy for improving extraction quality across heterogeneous AMA tasks.
- Methodologically robust evaluation protocol: We design a role-separated evaluation process that prevents LLMs from scoring their own outputs, using blinded, model-agnostic comparisons to ensure reliable and unbiased assessment of extraction quality.
- Task-specific automation guideline: Drawing on empirical performance patterns, we propose a three-tier classification of meta-analysis information types based on their suitability for automation and tolerance for error, offering practical guidelines on when to automate, when to review, and when human judgment remains essential.

## 2 Methodology

**Figure 1:**
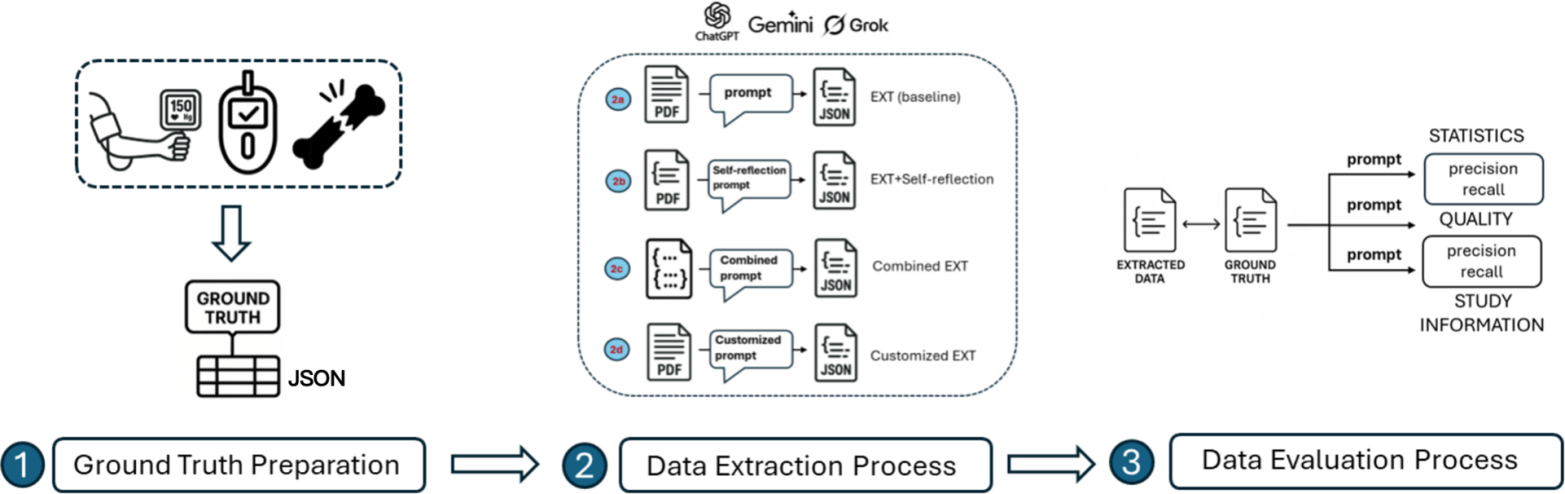
Overview of the whole workflow. Full-text RCTs were collected from published meta-analyses and annotated to construct a ground-truth dataset, which served as the basis for evaluating and comparing multiple extraction methods. Figure 1 Long description.

With LLMs at the center of our methodology, our experimental design sought to answer the following questions: How reliable are generic LLM prompts at accurately and completely extracting relevant data from diverse fields covered by potential meta-analyses? If we follow up the original LLM prompt with a subsequent prompt, asking the LLM to revise and improve upon its initial data extraction, to what degree will the accuracy improve? Are we able to improve the overall accuracy of the data extraction if we combine the outputs of several different LLMs on the same task? And finally, if we move beyond generic prompts to more domain-specific and customized prompts for each targeted meta-analysis field, are we able to achieve greater accuracy? Figure 1 provides an overview of the empirical design, from ground-truth construction to extraction and evaluation. The following sections describe each stage of this process, beginning with the construction of the ground-truth dataset for benchmarking.

### 2.1 Study selection and ground-truth preparation

We sought to evaluate the accuracy and generalizability of our approach by replicating the datasets used by six diverse meta-analyses (MA1–MA6) published between 2021 and 2025, whose underlying data we aimed to reproduce through our automated data extraction technique. We selected two studies from each of the three medical areas: hypertension (pharmacist-led care and diet interventions), diabetes (blood sugar control strategies and nutrition effects on insulin), and orthopaedics (bone fracture treatment and pharmacological impact on bone metabolism). These areas were chosen because they represent broad levels of complexity in how treatments and results are described in the papers. All were published in top-tier journals (Q1 or Q2). From each meta-analysis, we acquired and reviewed the full texts and identified the underlying RCTs studies, resulting in a total of 58 original manuscripts to serve as the testbed for automated data extraction. Table 1 shows the characteristics of the six meta-analyses whose underlying data we attempted to reproduce via LLMs applied to the original 58 papers.

**Table 1:** Characteristics of the included meta-analyses Table 1 Long description.

	Author		No. of		Min/Max
No.	(Year)	Field	RCTs	Primary outcome(s)	tokens used
MA1	Matsumoto et al. (2024)^25^	Hypertension	13	Systolic blood pressure	1033/3871
MA2	Guo et al. (2021)^26^	Hypertension	10	Systolic and diastolic blood pressure Anthropometric measures (weight, BMI, and waist circumference) Metabolic indicators (fasting glucose, total cholesterol, triglycerides, LDL-C, and HDL-C)	1549/3871
MA3	Khalid et al. (2023)^27^	Diabetes mellitus	10	Lipid profile components (TC, TG, LDL, VLDL, and HDL)	1291/3097
MA4	Yu et al. (2025)^28^	Diabetes mellitus	6	HOMA-IR	1549/2581
MA5	Kim et al. (2024)^29^	Orthopedic	7	BMD (femoral neck, total hip, and lumbar spine) BTM (CTX, P1NP, bone ALP, and Osteocalcin)	775/3613
MA6	Oldrini et al. (2022)^30^	Orthopedic	12	Functional Outcomes (DASH, PRWE, and EQ-5D) at 3 and 12 months Range of motion (flexion, extension, pronation, and supination) at 3, 6, and 12 months Grip Strength (% of the contralateral side) at 3, 6, and 12 months Radiological parameters (palar tilt, radial inclination, ulnar variance, and step-off) in immediately post-surgery and at more than 3 months.	1291/3355

To create the ground-truth dataset, we manually extracted all the required data from the 58 RCTs and converted the dataset into a structured JSON format (Step 1 in Figure 1). Importantly, we did not use data copied directly from the published meta-analyses, instead, we relied only on data directly extracted from the original RCT texts to ensure the ground truth was independently derived. We only extracted the specific measurements and outcomes that were actually used in the quantitative analyses of each meta-analysis, making sure our reference data matched what was actually synthesized. Two trained research staff independently reviewed and checked the JSON outputs against the analysis targets. We also drew on medical experts to validate our selection and interpretation of key variables. Any disagreements or unclear points were worked out through discussion. This expert-reviewed, agreed-upon dataset became our gold standard for testing how well the LLM-based extraction performed.

### 2.2 LLM-based extraction process

We evaluated the data extraction performance of three LLMs: *GPT-4o-mini* (OpenAI, released July 2024),^31^ *Gemini-2.0-flash* (Google DeepMind, released February 2025),^32^ and *Grok-3* (xAI, released April 2025),^33^ which we refer to henceforth simply as GPT, Gemini, and Grok, respectively. The evaluations were conducted in April 2025. We chose these models based on their reported capabilities^34^ ^–^ ^38^ for various data extraction tasks, including information from medical literature, as well as evidence synthesis, together with practical considerations like cost and API availability. Among them, we deliberately chose GPT-4o-mini, a more efficient variant of OpenAI’s flagship models like GPT-4 and GPT-4o, to explore whether a smaller, more affordable model could still deliver strong performance in data extraction. This decision reflects our interest in understanding the trade-offs between model size, cost, and accuracy in real-world meta-analytic workflows. The extraction followed a structured four-step prompting pipeline, and all models were required to return responses in a structured JSON format. Each full-text RCT manuscript was processed directly by a chosen model as a PDF file without any preprocessing. Token analysis confirmed that all documents were well within each model’s maximum context window, ensuring the robustness of the experiments. As shown in Table 1, all PDF files were with the 4,000 token limit, enabling complete document input without truncation or splitting. To minimize output variability, all documents were processed using the same fixed prompting pipeline, with no variation across inputs. Each model inference was conducted with the temperature set to 0 to maximize determinism. During preliminary testing, we re-ran identical prompts on the same documents and found that the outputs were highly consistent under these conditions. Minor differences, when present, were limited to formatting aspects (e.g., JSON field ordering) and did not affect the extracted content. All prompt templates used in this process are provided in the Supplementary Material. We designed four distinct data extraction strategies using all three LLMs depicted in Step 2 in Figure 1 follows:

*Step 2a: Baseline extraction (EXT):* The initial experiment served as a baseline against which more refined approaches could be benchmarked. In this experiment, we designed a detailed but generic prompt which was medical-domain agnostic, with the instruction to extract the relevant study characteristics and outcome data. The objective was to extract relevant study characteristics and outcome data from each full-text RCT manuscript. For each extracted field, the model was also required to (i) assign a confidence level (high, medium, or low) and (ii) specify the source section within the full text from which the value was acquired. This was implemented as a safeguard against model hallucination and to promote traceability. The prompt clearly articulated the need for factual responses without invoking its internal inference or estimation. We refer to this baseline extraction output as *EXT*.

*Step 2b: Self-reflection and revision (EXT+Self-reflection):* In the second step, we instructed the model to review its own previous output through a process called “self-reflection.”^39^ This process involves re-evaluating the initial extraction in order to identify and correct potential errors, as well as to revise confidence levels where appropriate. To enable this, the initial extraction results from Step 2a were provided back to the same model, along with the original PDF document and a structured prompt requiring it to reflect on its first response. Self-reflection is a common approach for mitigating hallucination that considered effective in other tasks where models apply complex reasoning or multi-step thinking.^40^ ^,^ ^41^ For meta-analysis, where important information is often scattered throughout a paper or buried in less obvious sections, the hypothesis was that this step may assist in locating omitted data points while correcting others. For each revision, the model was required to provide the original value, the new value, and a brief explanation of why it made the change. We refer to the outputs from this step *EXT+Self-reflection*.

*Step 2c: Combined extraction (Combined EXT):* Since all models have different capabilities and biases, our next hypothesis sought to determine if it is possible to improve the overall data extraction accuracy by combining the baseline responses of all three LLMs. The approach is based on ensemble learning theory, which states that combining outputs from different models, especially when they have complementary strengths, can enhance overall performance.^42^ ^,^ ^43^ Recent studies have confirmed that ensemble methods combining outputs from multiple LLMs can better extract biomedical concepts from clinical text and improve tasks like identifying medical terms.^44^ ^,^ ^45^ We forwarded the baseline EXT outputs from GPT, Gemini, and Grok to a merging process. The merging process was performed by another LLM (Gemini), which was guided by a structured prompt containing rule-based instructions. The model was instructed to strictly follow the rules instead of creating new data points or making inferences. The merging rules followed a hierarchical logic: (1) if two models agreed on a value and the third differed, the majority value was retained; (2) if all three values were different, the value associated with the highest confidence score was selected (when available); (3) in the absence of confidence scores, the most complete and internally consistent value was chosen, based on predefined criteria, such as field completeness and conformity to expected data types; and (4) for nested fields (e.g., outcome measures or participant details), the same hierarchical approach was applied recursively. The prompt strictly prohibited any rewording, subjective commentary, or fabrication of data. Importantly, the merging LLM operated solely on the EXT outputs without access to the model identities or the original document content. This ensured that the merging process remained decoupled from the original extraction logic and unbiased by the specific characteristics of any single model. We call the final merged outputs *Combined EXT*.

*Step 2d: Customized extraction (Customized EXT):* Given the proclivity of LLMs to display responses in highly variable levels of accuracy and quality depending on the detail and structure of the prompts, we hypothesized that accuracy gains might be achievable by tailoring the prompt to the specific medical domain relevant to the study. Therefore, we created a set of domain-specific prompts, customized to match the topic of each meta-analysis (e.g., hypertension, diabetes, and orthopaedics). These prompts were customized to reflect the specific outcomes and variables prioritized by the authors of each meta-analysis. For example, when the prompt primed the LLM to assume expertise in a field like “orthopedic and metabolic bone disease,” the LLM was explicitly instructed to attend to the domain-relevant data extraction with the guidance to: “*Focus on these outcomes: Bone Mineral Density (femoral neck, total hip, and lumbar spine) and Bone Turnover Markers (CTX, P1NP, BONE ALP, and Osteocalcin)*.” The customized prompts were applied across the same three LLMs (GPT, Gemini, and Grok), and the outputs we call as *Customized EXT*.

### 2.3 Evaluation

All extraction outputs (EXT, EXT+Self-reflection, Combined EXT, and customized EXT) were evaluated against the ground truth at the field level. To reflect how extracted data are used in real-world meta-analyses, we organized the evaluation into three functional categories: (1) *statistical results*, which included sample sizes, interventions or comparison details, and different outcomes, including mean, standard deviation, effect sizes, confidence intervals, *p*-values, adverse events, and dropouts; (2) *quality assessment*, covering risk of bias domains, such as randomization, allocation concealment, and blinding; and (3) *basic study information*, including basic study information, such as title, author, years, population characteristics, and eligibility criteria. These categories were derived from the structure of the model-generated JSON outputs. The ground-truth dataset was organized into the same three categories, allowing direct comparison between model outputs.

#### 2.3.1 LLM-based evaluation and human validation

We employed the LLM (Gemini) to perform file-level evaluation to efficiently assess extraction quality. The evaluation was designed to be unbiased and reliable by structuring it as a series of simple comparisons between extracted outputs and their corresponding ground-truth values, formatted as JSON key–value pairs. To ensure objectivity, Gemini received only the extracted value and the matching ground truth for each field. It did not have access to the original prompt, full document, or information about which model had produced the extraction. This setup was specifically intended to prevent evaluation bias, such as a model validating its own output, and to ensure that the assessment was based solely on semantic equivalence between extracted and reference values. For each field, the model was instructed to assign one of three evaluation labels: (1) *Correct*: The extracted value matched the ground truth in meaning. (2) *Missing*: The ground truth included a value, but the model failed to extract it. (3) *Hallucinated*: The model extracted a value that was not present or justifiable in the original text. For hallucinated fields, we implemented a secondary classification to better understand the nature of the error. These were broken down into: (1) *incorrect value*, where the extracted field was factually wrong; (2) *incorrect unit*, where the number was right but the unit was wrong; and (3) *overgeneralized*, where the extracted information information was too vague, non-specific, or lacked necessary detail/context. All evaluations used a temperature of 0.0 to ensure guarantee deterministic outputs. This approach enabled scalable, reproducible, and fine-grained assessment of extraction performance across thousands of data fields.

To check whether this LLM-based evaluation was reliable, we conducted a blinded manual review of 900 sample fields. Using stratified random sampling, we picked up to 30 fields from each combination of model (GPT, Gemini, and Grok), extraction method (EXT, EXT+Self-reflection, Combined EXT, and customized EXT), and data category (statistics, quality, and information). Two independent reviewers, who did not know the LLM-assigned labels or each other’s responses, labeled each field as Correct, Hallucinated, or Missing by comparing it to the ground truth. Agreement between human reviewers and LLM-assigned labels was 96.09%, and agreement between the two human reviewers was very high (Cohen’s κ=0.987 ), showing that the automated evaluation approach offers a reliable and efficient alternative to manual labeling for large-scale evidence extraction tasks.

#### 2.3.2 Performance assessment

We calculated *precision* and *recall* at the element level to measure how well the data extraction worked. Precision shows what proportion of extracted elements were correct, while recall shows what proportion of relevant elements from the ground truth were successfully found. We calculated these measures separately for each category and extraction method, giving us complementary views of how accurate and complete the extraction was. Then, we compared both the effectiveness of different extraction strategies and how well different models performed. A non-parametric Friedman test was conducted across task categories to assess whether the observed differences in performance were statistically significant, with a *post-hoc* Nemenyi test used to identify differences. Based on this comparison, we identified the method with the best overall performance and then used it as a foundation for comparing models. Within this method, we ranked models by their recall scores, and calculated their *mean rank* to provide a summary measure of extraction quality.

## 3 Results

We present the evaluation results from three different angles. First, we look at overall performance, comparing different methods and models and examining how they interact with each other. Next, we evaluate performance differences across individual meta-analysis datasets to identify any trends specific to particular medical areas. Finally, we examine the distribution of different types of data extraction errors, both overall and in relation to specific fields and models.

### 3.1 Overall performance

Table 2 shows precision and recall for each method–model combination, where data extraction was evaluated and averaged across all 58 papers and broken down by different data types (i.e., statistics results, quality assessment, and basic study information). Overall, extraction performance varied substantially by field type, especially in recall, while being relatively high in precision. Study information fields were generally extracted most completely (recall ranging from 0.52 to 0.84), followed by quality assessments (recall ranging from 0.7 to 0.78), while statistical results proved to be the most challenging (recall ranging from 0.21 to 0.76). The table also shows that the customized EXT approach outperformed others across all three categories of data type extractions by a clear margin.

**Table 2:** Overall performance for different extraction approaches across models Table 2 Long description.

Approach	Model	Precision	Recall	Recall rank
*Statistics results*				
EXT (Baseline)	GPT	0.856	0.214	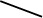
	Gemini	0.911	**0.445**	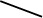
	Grok	**0.939**	0.389	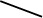
EXT+Self-reflection	GPT	0.854	0.231	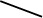
	Gemini	0.905	0.441	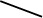
	Grok	**0.928**	**0.464**	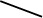
Combined EXT	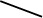	0.906	0.430	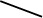
Customized EXT	GPT	0.814	0.381	3.0
	Gemini	**0.952**	**0.760**	1.0
	Grok	0.921	0.697	2.0
*Quality assessment results*				
EXT (Baseline)	GPT	0.866	0.730	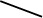
	Gemini	0.914	0.702	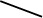
	Grok	**0.936**	**0.760**	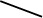
EXT+Self-reflection	GPT	0.878	0.744	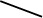
	Gemini	0.928	0.739	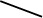
	Grok	**0.933**	**0.763**	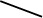
Combined EXT	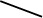	0.927	0.759	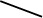
Customized EXT	GPT	0.863	0.730	2.0
	Gemini	0.920	0.727	3.0
	Grok	**0.953**	**0.782**	1.0
*Study information results*				
EXT (Baseline)	GPT	0.770	0.521	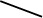
	Gemini	0.870	0.631	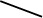
	Grok	**0.913**	**0.754**	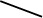
EXT+Self-reflection	GPT	0.764	0.523	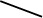
	Gemini	0.864	0.646	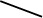
	Grok	**0.931**	**0.756**	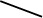
Combined EXT	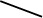	0.887	0.705	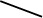
Customized EXT	GPT	0.745	0.760	3.0
	Gemini	0.845	0.808	2.0
	Grok	**0.887**	**0.841**	1.0

#### 3.1.1 Extraction method comparison

**Figure 2:**
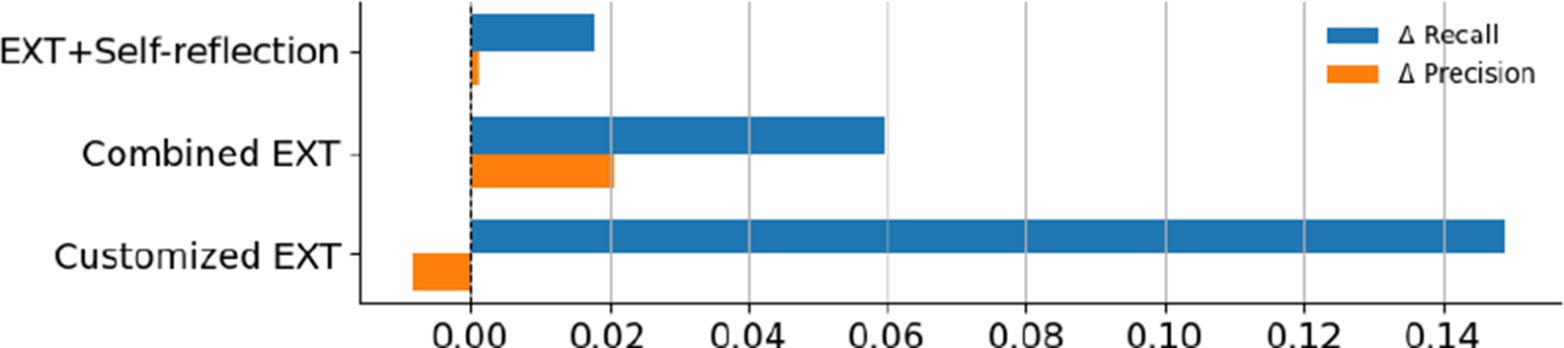
Average performance change ( Δ precision and Δ recall) of three extraction strategies relative to the EXT baseline.

**Figure 3:**
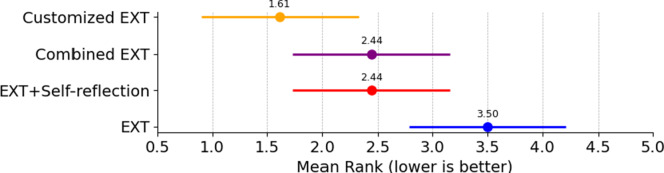
Friedman–Nemenyi critical difference (CD) graph based on mean rank in recall. If the two horizontal line segments in the figure do not overlap, it signifies a significant performance difference between the two methods. Figure 3 Long description.

Here, we quantify the degree to which each of the extraction method variants improved over the baseline EXT. Figure 2 summarizes the average change over the EXT in recall and precision for each method. Customized EXT delivered the biggest recall increase, with an average gain of 14.8% across data categories and models. This came with a tiny precision drop ( − 0.8%), showing a controlled trade-off between finding more relevant content and slight over-extraction. Combined EXT offered the most balanced results, achieving a solid recall gain (5.9%) plus a small precision increase (2.0%). This suggests that combining multiple models can improve both completeness and accuracy in a stable way. Self-reflection EXT showed only minor improvements in both recall (1.8%) and precision (0.1%), suggesting that while instructing models to review and revise their responses may lead to more reliable outputs, it does not significantly aid with retrieving additional relevant information. A Friedman test confirmed that the recall differences between methods were statistically significant ( $\chi^{2}$ (3) = 9.81, *p* = 0.0203), showing that an extraction strategy does have an effect on retrieval performance. The follow-up Nemenyi test found that EXT was significantly outperformed by customized EXT, though the difference between EXT and Combined EXT was not statistically significant (Figure 3). Combined EXT and EXT+Self-reflection showed similar overall recall performance, with matching mean ranks (2.44), suggesting their extraction effectiveness is nearly identical. Mean ranks were used to summarize how each extraction method performed across all model–task combinations. For each combination, methods were ranked based on their recall scores, with higher recall receiving better (i.e., lower) ranks. These ranks were then averaged to produce a mean rank for each method.

#### 3.1.2 LLM model performance comparison

To compare the capabilities of the LLM models for the data extraction task fairly without confounding factors from different extraction methods, we used customized EXT (which performed best overall) as the shared extraction baseline. For each of the three task categories, we ranked the models by recall, which are reported in Table 2. Then, we further averaged each model’s recall ranks across the three task categories to give mean ranks. The resulting mean ranks (although not shown in Table 2) revealed that Grok achieved the best overall performance (1.3), followed by Gemini (2.0) and GPT (2.7).

For the extraction of the statistical results category data, Gemini performed best (average rank 1.0), showing its strength with numerical data. Grok came second (2.0), while GPT lagged behind (3.0), suggesting smaller models struggle more with numerical content. For quality assessment, Grok ranked highest, GPT came second, and Gemini came third. Quality assessment fields often use less structured, more subjective language that sometimes spans multiple sentences, making them harder to extract reliably. While all models showed some variability here, Grok indicated stronger capabilities at handling loosely structured content. For study information data extraction (e.g., structured metadata like titles, authors, years, population details, and eligibility criteria), Grok again performed best (1.0), followed by Gemini (2.0) and GPT (3.0). This data category was evidently less challenging for all models, presumably due to this type of data tending to be represented using predictable wording and formatting. Grok depicted the best performance by significant margins, perhaps suggesting that, with it being the newest model, there are increased data capture capabilities in the more advanced models on these types of tasks. Across all data type categories, Grok displayed the most reliable data extraction performance, Gemini excelled with complex or variable fields, while GPT lagged repeatedly behind the others. This confirms that model differences meaningfully affect extraction quality, especially when content varies in structure and clarity.

#### 3.1.3 Comparison of method–model combinations

All three models improved their recall when using the Customized EXT method (Table 3). Gemini realized the biggest boost (17.2%), followed by Grok (13.9%) and GPT (13.5%). In contrast, EXT+Self-reflection produced only small recall changes, with modest increases for Grok (2.7%), Gemini (1.6%), and GPT (1.1%). Combined EXT showed strong improvements for GPT (14.3%) and a modest gain for Gemini (3.8%), but slightly decreased Grok’s performance ( − 0.3%). Precision changes were generally small without clear patterns, though GPT did exhibit a notable precision increase (7.6%) with the Combined EXT method. These results indicate that generating more tailored and customized data extraction prompts can effectively improve recall, while strategies that leverage ensemble-based solutions for data extraction can provide balanced improvements in both recall and precision for certain models like GPT.

**Table 3:** Comparison of model performance across different methods Table 3 Long description.

Model	Method	Δ Recall	Δ Precision
GPT	EXT+Self-reflection	0.011	0.001
	Combined EXT	**0.143**	**0.076**
	Customized EXT	0.135	− 0.023
Gemini	EXT+Self-reflection	0.016	0.001
	Combined EXT	0.038	**0.008**
	Customized EXT	**0.172**	0.007
Grok	EXT+Self-reflection	0.027	**0.001**
	Combined EXT	− 0.003	− 0.023
	Customized EXT	**0.139**	− 0.009

### 3.2 Performance across meta-analyses

Previous sections examined data extraction performance at the level of overall averages by data types, here we provide a deeper and more granular analysis, examining how different extraction methods performed across the six meta-analyses RCTs’ datasets (MA1–MA6) as well as data types, in order to surface the existence of performance variabilities. Each meta-analysis RCTs’ dataset varied in structure and field density, offering a useful perspective on the capability of each method.

#### 3.2.1 Data extraction method comparison

**Figure 4:**
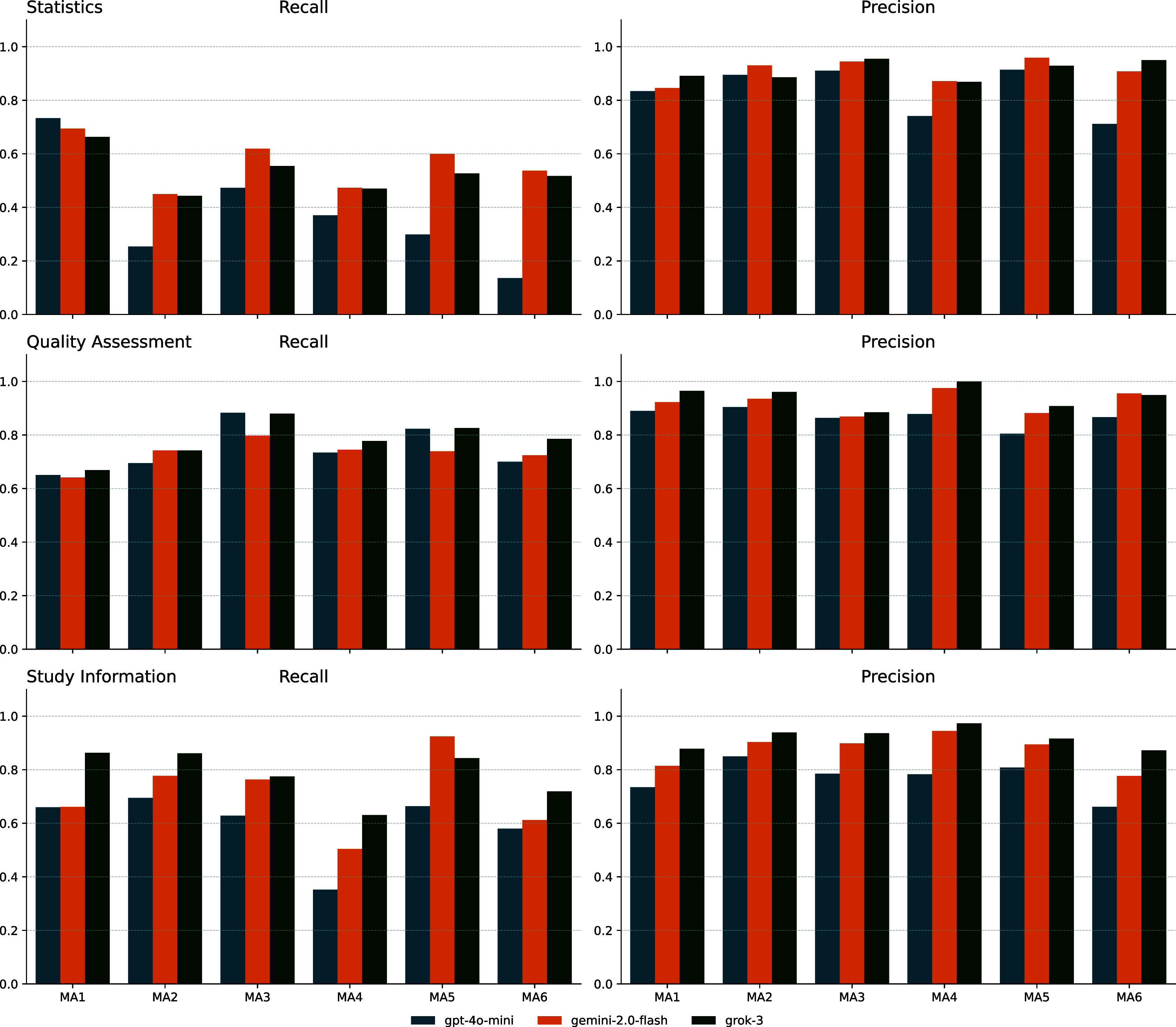
Methods comparison across meta-analyses. Figure 4 Long description.

As shown in Figure 4, the performance of the different extraction methods varied significantly across different MA datasets. For statistical data extraction, methods struggled with MA datasets like MA3, MA4, MA5, and MA6, which contained high levels of specialized medical terms (e.g., lipid profiles and bone turnover markers) and complex structures such as multi-level groupings. In these challenging cases, Customized EXT achieved recall gains over 20% compared to baseline methods, likely because it was already primed in the prompt for the specialized context and could thus better recognize specialized field terms. Study information fields also showed wide variations in recall, with Customized EXT significantly outperforming other approaches across most datasets. While almost all methods handled structured metadata like titles, authors, and years effectively, extracting population characteristics proved much harder. These details were often buried in narrative text without standard wording. In real meta-analyses, the population details authors report vary depending on their study’s focus—some emphasize demographics, others clinical history—making it challenging to use a single extraction approach or to provide guidance to LLMs in explicit prompt instructions. Customized EXT performed better by collating and drawing together scattered mentions of relevant data points through the manuscripts to build complete study information descriptions, while baseline methods often captured only fragments. For quality assessment, performance differences between methods were less dramatic. Since this information is typically clearly labeled with familiar terms (like “blinded” or “random sequence generation”), even simpler extraction methods achieved relatively high recall and precision. Combined EXT retrieved the most fields in most datasets for this category (MA1, MA2, MA3, and MA6), showing that mixing multiple extraction inputs worked effectively for typical quality assessment content structure in published papers. EXT+Self-reflection provided little extra value. Precision stayed largely stable across all methods and datasets.

#### 3.2.2 LLM model comparison

**Figure 5:**
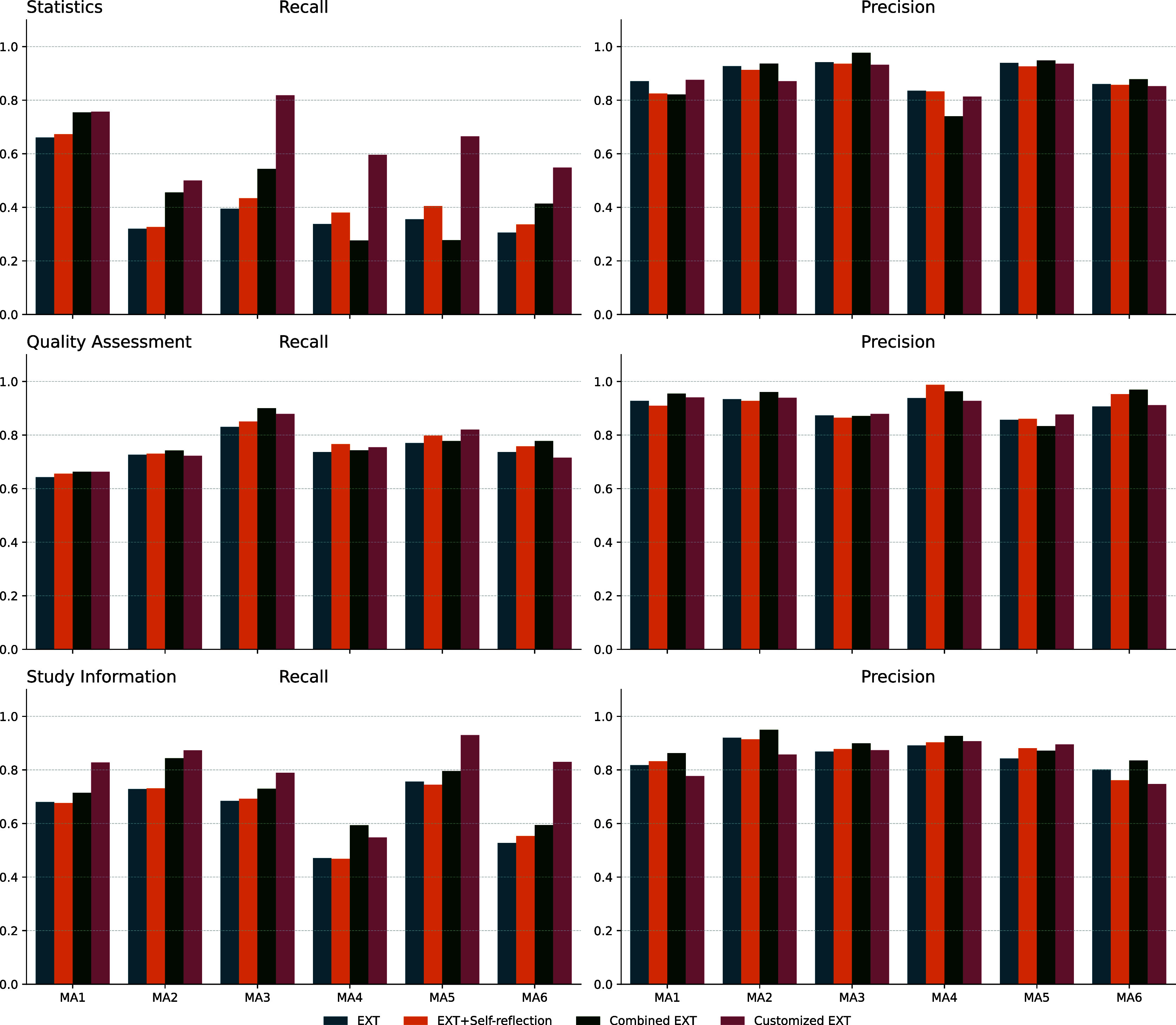
Models comparison across meta-analyses. Figure 5 Long description.

We also assessed the strengths of different models from the three task categories. As shown in Figure 5, the statistics category showed the biggest differences in model performance for recall. Grok and Gemini outperformed GPT, with Gemini achieving the highest recall across most datasets (MA2, MA3, MA4, and MA5). These datasets included scattered numerical data, technical terms, and specialized abbreviations (like LDL-C and BTM), which likely made extraction difficult for less capable models like GPT. GPT’s recall typically stayed below 0.5, dropping to around 0.16 in MA6. All three models maintained high precision (typically above 0.75), showing that better recall did not lead to extracting irrelevant data. For quality assessment fields, model performance was similar. All models achieved comparable recall and precision across datasets, likely because these fields use standardized formats across studies. Small differences appeared, such as slightly better recall for GPT in MA3 and MA5, and near-perfect precision for Grok in MA4. The study information category also showed notable differences in model performance, particularly for recall. Grok performed best in most datasets except MA5. Gemini also extracted fields effectively across datasets, achieving the highest recall of 0.96 in MA5, while GPT performed less well. Precision remained stable across models, though GPT showed slightly lower precision compared to Gemini and Grok across all datasets.

#### 3.2.3 Comparison of method–model combinations

We analyzed how the most effective model–method combinations were affected by data category, identifying the configuration with the highest recall for each meta-analysis (Table 4). In the statistics category, Gemini paired with Customized EXT achieved the highest recall in four of six datasets (MA2, MA3, MA5, and MA6), while Grok led in MA4, and GPT performed best in MA1. This shows that for complex statistical data, the model’s capabilities matter more than the prompting method, even against advanced data extraction approaches. For quality assessment, Grok was the most frequent top performer, especially when paired with either Customized EXT or EXT. Notably, the best result in MA3 came from Combined EXT using model aggregation, suggesting that blending outputs from multiple models can help when field boundaries are not clearly defined. Precision remained high across all setups (often above 0.95), reflecting the more predictable structure of these fields. For study information, Customized EXT delivered the best performance in most datasets except MA4, indicating its strength in handling descriptive and fragmented fields like population characteristics or eligibility criteria. However, the best model varied by dataset: Grok excelled in MA1, MA4, and MA6, while Gemini led in MA2, MA3, and MA5. The absence of GPT among top performers in this category may suggest that older models struggle with study details, where understanding context and combining information from multiple sentences is essential.

**Table 4:** Best-performing model–method combinations by category Table 4 Long description.

Dataset	Method	Model	Precision	Recall
*Statistics results*				
MA1	Customized EXT	GPT	0.840	0.792
MA2	Customized EXT	Gemini	0.947	0.608
MA3	Customized EXT	Gemini	0.927	0.893
MA4	Customized EXT	Grok	0.970	0.674
MA5	Customized EXT	Gemini	0.986	0.799
MA6	Customized EXT	Gemini	0.972	0.785
*Quality assessment results*				
MA1	Customized EXT	Grok	1.000	0.684
MA2	Customized EXT	Grok	0.962	0.773
MA3	Combined EXT	Combined	0.871	0.900
MA4	EXT	Grok	1.000	0.778
MA5	Customized EXT	Grok	0.935	0.878
MA6	EXT	Grok	0.955	0.810
*Study information results*				
MA1	Customized EXT	Grok	0.826	0.919
MA2	Customized EXT	Gemini	0.841	0.894
MA3	Customized EXT	Gemini	0.903	0.829
MA4	EXT+Self-reflection	Grok	0.980	0.639
MA5	Customized EXT	Gemini	0.926	0.965
MA6	Customized EXT	Grok	0.842	0.882

### 3.3 Error distribution

While overall performance metrics like recall and precision show how well each method works, they do not fully reveal where and what types of errors occur. To better understand the limitations of different data extraction approaches, we investigated how different types of errors were distributed across specific fields and models. This section breaks down the error distribution with a focus on general trends. Additional analyses, including field-specific differences and the interaction between models and methods, are presented in the Supplementary Material for further reference.

#### 3.3.1 Overall analysis

We analyzed how errors were distributed across all extracted fields. Of all error cases we analyzed, the majority—87.8% (19,470 instances)—were missing fields. This strongly indicates that current methods and LLM combinations are still challenged to reliably find and extract all relevant fields for fully automating meta-analyses, especially when information is buried in narrative text, spread across multiple sentences and paragraphs, or expressed in non-standard ways. The next most common error was incorrect values, comprising 10.3% (2,296 instances). These typically involved wrong numerical data, misaligned subgroup details, or misinterpreted statistical results (e.g., reporting the control group sample size as 32 instead of the correct value of 17, reporting 10% male in total instead of 5% in the intervention group, or listing separate BMD values instead of the correct combined mean). Less common were overgeneralized errors (e.g., omitting “living independently” from inclusion criteria, or replacing a detailed randomization method such as “1:1 computer-generated list” with the generic term “randomized”) at 1.2% (267 instances) and incorrect unit errors at 0.7% (163 instances). From this overall error distribution, we can see that detecting fields (recall) remains the main challenge in current extraction processes, while precision issues, though important, account for a smaller portion of errors. For more detailed analysis, please refer to Tables 1 and 2 in the Supplementary Material.

## 4 Discussion

This study evaluated how well LLMs perform structured data extraction for AMA, aiming to identify their current capabilities and key weaknesses in practical, cross-domain situations. We summarize the main findings here, elaborate on the implications, and present a recommendation guideline based on our results for guiding researchers on how to mitigate risks when using LLMs for the automation of data extraction in meta-analysis.

### 4.1 How reliable are current LLMs for automated structured data extraction in MAs?

Current state-of-the-art commercial LLMs offer only partial reliability for structured data extraction in meta-analyses. While they show consistent performance on simpler study-level characteristics and some risk-of-bias items, their extraction of statistical results remains error-prone and incomplete, making them unsuitable as standalone tools for end-to-end evidence synthesis without human intervention. These findings highlight the need to carefully examine where LLMs succeed or struggle across different aspects of data extraction, including common errors, data type differences, prompting strategies, model variation, and implications for practical use.

#### 4.1.1 What are the most common data extraction errors?

The frontier commercial LLM models we investigated in this study most commonly made errors of omission rather than commission. While precision remained high across different LLMs, recall suffered due to missing or partially extracted fields, especially when dispersed or embedded in complex text. Incorrect values were the next major issue, often reflecting misinterpretation of group-specific statistics. Overgeneralization and unit errors were rare but indicate challenges in preserving specificity and format. Overall, recall limitations remain the primary obstacle to reliable automated data extraction.

#### 4.1.2 How reliable are LLMs at extracting different types of data categories?

Extraction reliability depended on the data categories. Structured, numerical fields, such as statistical outcomes, were easier for models like Gemini to extract. In contrast, quality assessments and study characteristics required more contextual reasoning, where Grok performed better. Grok achieved the highest recall and precision in those areas. GPT consistently lagged behind in all three categories. Its data extraction performance led to fewer incorrect values but also much lower recall, especially in complex fields. These differences highlighted that no single model performed equally well across all meta-analytic tasks. Each LLM had unique strengths and is therefore better suited to specific types of information or evidence-synthesis tasks.

#### 4.1.3 To what extent do different prompting strategies affect performance?

Prompting strategies significantly influenced extraction quality. Self-reflection led to small gains in recall, usually only 1–2 percentage points. It offered a possible way to recover hard-to-detect content with minimal human involvement, but to work well, it must be narrowly focused. It was more helpful for refining extractions in ambiguous or subjective fields like risk of bias, but had little effect when the original output was already strong. Combining outputs from different models gave more reliable improvements, raising recall by about 5% on average. Combining their outputs helped build a more complete picture, especially in cases where no single model captured everything on its own. Meta-analysis needed both completeness and reliability, this strategy offered a practical fallback. Model disagreement helped identify uncertain cases for review, while agreement strengthened confidence in the result. Rather than relying on a single system, combining outputs provided a flexible way to improve performance without requiring complex fine-tuning. Domain-specific prompts had the strongest effect. By focusing the model on important fields (e.g., different medical domains, baseline characteristics, and trial outcomes), recall improved, especially in more complex categories like statistical outcomes. However, in some cases, this led to a slight drop in precision, as models included broader or less certain information. This trade-off is common in extraction tasks^46^ ^,^ ^47^ and should be managed depending on whether completeness or accuracy is more important. Customized prompting offered a low-cost, adaptable way to raise extraction quality without altering the underlying model.

#### 4.1.4 How variable are the performances of different cutting-edge LLMs?

GPT tended to extract less data but generated relatively few mistakes. Grok focused on accuracy and achieved the highest precision in statistical extraction (0.939), though with limited recall. Gemini, by contrast, offered a more balanced outcome. It accepted some uncertainty in exchange for broader extraction, resulting in moderate recall while keeping precision within a reasonable range. This approach produced a more complete dataset at the cost of a slight drop in accuracy, which is an acceptable trade-off when a full evidence capture is required. In some ways, Gemini exhibited patterns akin to a human reviewer, scanning widely and using context to pick up subtle clues throughout the article.

#### 4.1.5 What does this mean for practice?

Customized prompting emerged as the most promising strategy for improving structured data extraction with LLMs. By matching prompts with highlight fields most relevant to meta-analytic synthesis, such as statistical outcomes, LLMs were better guided toward useful outputs. This approach consistently improved recall with only tiny drop in precision, making it particularly valuable for tasks where completeness is essential. Used thoughtfully, task-specific prompts moved LLM-based extraction closer to real-world applications, helping bridge the gap between promising model performance and the demands of AMA. In addition, model selection should align with the nature of the task.^48^ ^,^ ^49^ Gemini might be more effective for capturing broader statistical data, Grok was better suited for analysing quality assessment or reviewing protocols. GPT, while more limited, might still be useful in fast, low-risk screenings or as part of a larger system that includes human oversight. Combining outputs from multiple or integrating the reflection step, further enhanced performance in some cases, especially for weaker base models. However, even with these improvements, human oversight remains necessary, which could help build workflows that were both fast and trustworthy, especially for large-scale or time-sensitive reviews.

### 4.2 What level of automation is enough? Recommendations for meta-analytic extraction

**Figure 6:**
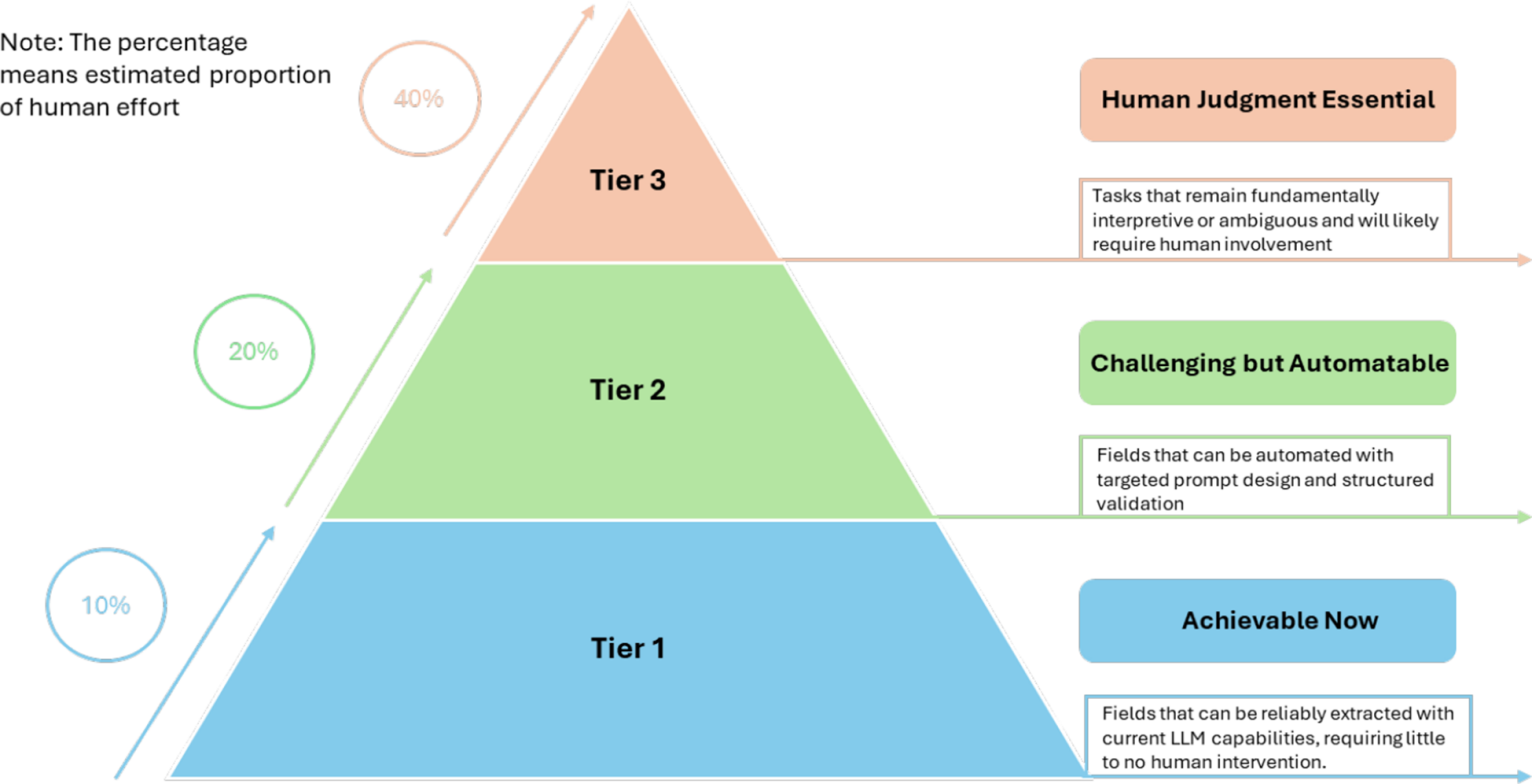
Three-tier automation guideline for structured data extraction in meta-analysis, based on task difficulty, error risk, and need for human oversight. Percentages on the left indicate estimated proportions of total human effort required for each tier for verification of extracted data. Figure 6 Long description.

The concept of fully automated meta-analytic extraction imagines a system capable of capturing every relevant data point with near-perfect accuracy. But in practice, not all fields in a meta-analysis carry equal weight or require the same level of reliability to support valid conclusions. Some elements, such as statistical results, directly shape pooled estimates and clinical inferences, while others, like study context or author details, primarily serve descriptive or organizational purposes. Errors in the former category have a high risk to the integrity of meta-analytic conclusions, while inaccuracies in the latter may have a limited impact. Therefore, in conjunction with our findings, we propose structured, task-specific standards according to the different levels of automation in meta-analytic data extraction. These suggestions are not only technical, they reflect how real-world meta-analyses are read and used. Rather than aiming for perfection, extraction for AMA could be based on task-matched performance. We organize information types into three tiers, based on their suitability for automation, the extent of human judgment required and the risk associated with errors. As shown in Figure 6, these tiers represent a spectrum from routine, high-precision extraction (Tier 1), to tasks necessitating targeted oversight (Tier 2), to areas where interpretive reasoning is essential with maximal human-in-the-loop involvement (Tier 3).

*Tier 1—Achievable now* includes fields that are often clearly reported, structurally straightforward, and tolerant of minor errors. Examples include *study information*, such as population characteristics, trial location, and author details. These elements mainly aid filtering and contextual understanding. Because the risk of downstream impact from inaccuracies in these fields is low, our evaluations indicated that even basic prompting yields dependable performance (recall 72%–85%, precision 78%–98%), and reviewer effort could thus be limited to glancing checks of flagged items. These fields are well-suited for efficient, high-volume automation.

*Tier 2—Challenging but automatable* includes tasks such as *quality assessment*. These involve drawing inferences, understanding intent, and synthesizing incomplete information, which can be helped with semi-structured frameworks, such as RoB 2^50^ or GRADE.^51^ Errors in this tier carry moderate risk, especially if misjudgments affect inclusion criteria or bias assessments. LLMs can assist by retrieving relevant rationales and pre-filling fields. In this tier, human review is not optional but essential for accurate interpretation. Additionally, we found that LLMs, especially when guided by combined or reflective prompts, could help bring up relevant content. Using LLMs to pre-fill fields, point out supporting evidence, or mark uncertain judgments can be one way they significantly ease the workload for reviewers.

*Tier 3—Human judgment essential* includes fields central to meta-analytic synthesis, such as *statistical results* like effect sizes, confidence intervals, and heterogeneity measures. These demand high recall to capture all outcomes and high precision to avoid distorting effect estimates. Our experiments indicated that customized prompting could achieve moderate performance (recall approximately 76%, precision ranging from 81% to 92%), but even minor extraction errors in this domain could lead to substantial downstream bias. This tier involves the highest risk, as inaccuracies can directly compromise the validity of pooled analyses and clinical conclusions. As a result, tasks in this tier demand structured output formats, clear linkage to source text for verification, and mandatory human verification on primary effect estimates. Automation is feasible here, but only as part of a human-in-the-loop pipeline where reviewers retain responsibility for the final confirmation of each data point.

Table 5 summarizes these tiers, linking each information category to its role in meta-analysis, automation goals, reviewer strategy, and roadmap classification. This framework supports a modular AMA development path: starting with Tier 1 fields where performance is already effective, refining Tier 2 domains through task-specific improvements, and supporting Tier 3 tasks through assistive human-in-the-loop workflows. In this view, these results show that future development for AMA is best based on a modular extraction process, in which LLM outputs are directed to different post-processing steps depending on field type and confidence level. Progress toward AMA is less about reaching perfection everywhere, and more about knowing where “good enough” might be enough. This approach will enable AMA to advance step by step, beginning with categories that are already achievable, while refining others through focused model adjustments and task-specific training.

**Table 5:** Automation priorities, strategies, and roadmap tiers by information category Table 5 Long description.

Category	Role and sensitivity	Automation plan (target + reviewer strategy)	Roadmap tier
Statistical	Core to meta-analytic conclusions; errors distort effect estimates; low tolerance for mistakes	Recall ≥ 90%, Precision ≥ 95%. Use customized prompts to extract data with linked source text. Human reviewers must verify effect direction, completeness, and comparators.	Tier 3: Challenging; requires prompt tuning & manual validation
Quality assessment	Supports bias judgments; interpretive and semi-subjective; moderate-to-low consistency	Recall ≥ 85%, Precision ≥ 95%. Focus on rationale extraction. Use ensemble methods or self-reflection to retrieve supporting evidence. LLM may suggest labels, but the reviewer must interpret vague or implicit reporting. Human input is essential in unclear cases.	Tier 2: Automation feasible
Study information	Contextual metadata; often explicit; high tolerance for small errors	Recall ≥ 80%, Precision ≥ 90%. Use generic prompts or self-reflection to extract locations, population, and study details. Allow auto-accept unless low confidence. Review optional.	Tier 1: Achievable now with minimal oversight

### 4.3 Beyond extraction: Advancing dependable and ethical AMA

With the rapid advancement of LLMs, AMA has emerged as a key area in evidence synthesis. However, AMA extends beyond individual extraction tasks. It functions as a multi-stage system involving interconnected steps, from study identification and screening to knowledge synthesis.^52^ With well-defined prompts and clear input formats, LLMs have the potential to make extraction become a practical starting point for automation. Risk-of-bias assessment, while more interpretive, can be supported by a semi-structured framework, such as RoB 2^50^ or GRADE,^51^ with LLMs assisting in applying formal criteria and reducing reviewer variability. The most complex stage is evidence synthesis, involving selecting appropriate models (e.g., fixed- vs. random-effects), evaluating heterogeneity, performing sensitivity or subgroup analyses, and integrating findings with clinical context. Existing studies provide limited evidence on how LLMs can contribute to meta-analytic decision-making, not because it is unsuitable for automation, but due to unresolved theoretical and practical challenges.^52^ Recent developments in chain-of-thought and agent-based LLM systems indicate that these models may offer more capabilities for supporting complex synthesis tasks.^53^ ^,^ ^54^ Therefore, future efforts should focus on experimental applications like proposing sensitivity analyses and creating traceable analytical records to enhance transparency and reproducibility while allowing human experts to maintain authority over the core of evidence synthesis. The value of full AMA lies not in replacing expert judgment but in developing frameworks that make such judgments more traceable, repeatable, and transparent.

From an ethical perspective, LLMs in AMA raise concerns about how models present evidence. LLMs can produce seemingly authoritative outputs even with uncertain data,^55^ potentially causing users to place undue trust in conclusions. Though recent reasoning-capable LLMs show more observable logic, these steps don’t guarantee interpretability or validity,^56^ challenging evidence-based practice principles. AMA systems should incorporate transparency features,^57^ including uncertainty annotation, audit trails documenting output production, and model versioning for replication. Ethical use requires cautious automation that is open about processes and accountable to methodological standards.

Regarding methodological accuracy, we implemented careful measures to prevent feedback loops when using Gemini across multiple tasks. In the merging and evaluation stages, Gemini only operated on JSON-structured data without access to original texts or model identity. This design deliberately prevents the model from using prior knowledge of how values were generated. Additionally, our blinded human validation of 900 samples showed 96.1% agreement with the LLM-assigned labels, reinforcing the dependability of our evaluation framework. We selected Gemini for these roles not for uniformity but because it offered balanced precision–recall performance across information categories, making it an appropriate tool for non-inferential aggregation and scoring tasks.

### 4.4 Study limitations

This study has several limitations. First, although we evaluated LLMs on full-text RCTs, our dataset was drawn from a limited number of published meta-analyses across three clinical domains. Broader generalizability remains to be tested. Another limitation is that some key study data were embedded in charts, figures, or non-machine-readable graphical elements which presented an additional challenge. Future work will focus on delving deeper into exploring automated data extraction from these sources specifically. The models we tested were treated as black-box systems, without fine-tuning or access to intermediate model outputs. As a result, we could not directly diagnose why specific fields were omitted, or trace certain failure patterns back to model internals. Also, we did not systematically evaluate computational costs, API expenses, or processing times. Future work will also factor in these operational aspects. Newer models are continuously emerging with greater capabilities, and it is likely that the latest models leveraging test-time compute for increased “thinking” during inference, may achieve better performances than our results.

## 5 Conclusion

This study evaluated the structured data extraction capabilities of three state-of-the-art commercial LLMs for meta-analysis, comparing their performance across different data types and prompting strategies. While all models achieved high precision, recall remained limited due to the frequent omission of key information. Crucially, we found that customized prompts significantly improved data extraction quality, highlighting prompt engineering as essential for enhancing LLM effectiveness in real-world meta-analyses. Based on these insights, we developed clear, three-tiered guidelines for data extraction in meta-analysis, matching appropriate levels of LLM-based automation to specific tasks according to their complexity and error risk. Our results emphasized that effective automation of data extraction required not only capable models, but also clearly defined prompts and targeted human oversight.

## Supporting information

10.1017/rsm.2025.10066.sm001 Li et al. supplementary material Li et al. supplementary material

## Data Availability

All data analyzed in this study were extracted from published sources and are available in the article or supplementary material. Additional information is available from the corresponding author upon reasonable request.

## Supplementary material

The supplementary material for this article can be found at https://doi.org/10.1017/rsm.2025.10066.

## Long descriptions

### Figure 1 Long description

The flowchart is divided into three numbered sections.

Section 1, Ground Truth Preparation. At the top, a dashed box contains icons representing medical data: a blood pressure cuff, a glucose monitor, and a fractured bone. An arrow points down to a box labeled GROUND TRUTH, which is connected to a J S O N table icon.

Section 2, Data Extraction Process. This section is headed by logos for ChatGPT, Gemini, and Grok. It contains four parallel sub-processes labeled 2a through 2d.

- 2a: P D F icon to prompt to J S O N icon, labeled E X T baseline.

- 2b: P D F icon to Self-reflection prompt to J S O N icon, labeled E X T plus Self-reflection.

- 2c: P D F icon with brackets to Combined prompt to J S O N icon, labeled Combined E X T.

- 2d: P D F icon to Customized prompt to J S O N icon, labeled Customized E X T.

Section 3, Data Evaluation Process. A bidirectional arrow connects EXTRACTED DATA and GROUND TRUTH icons. An arrow points from this pair to three evaluation categories, each triggered by a prompt.

- STATISTICS: measuring precision and recall.

- QUALITY: measuring precision and recall.

- STUDY INFORMATION.

Large block arrows at the bottom indicate the sequential flow from stage 1 to 2 to 3.

Navigate back to Figure 1.

### Table 1 Long description

The table consists of six columns: No., Author Year, Field, No. of R C T s, Primary outcome s, and Min/Max tokens used.

Row 1: M A 1, Matsumoto et al. 2024, Hypertension, 13 R C T s, Systolic blood pressure, 1033/3871 tokens.

Row 2: M A 2, Guo et al. 2021, Hypertension, 10 R C T s, Systolic and diastolic blood pressure, Anthropometric measures weight, B M I, and waist circumference, and Metabolic indicators fasting glucose, total cholesterol, triglycerides, L D L-C, and H D L-C, 1549/3871 tokens.

Row 3: M A 3, Khalid et al. 2023, Diabetes mellitus, 10 R C T s, Lipid profile components T C, T G, L D L, V L D L, and H D L, 1291/3097 tokens.

Row 4: M A 4, Yu et al. 2025, Diabetes mellitus, 6 R C T s, H O M A-I R, 1549/2581 tokens.

Row 5: M A 5, Kim et al. 2024, Orthopedic, 7 R C T s, B M D femoral neck, total hip, and lumbar spine and B T M C T X, P 1 N P, bone A L P, and Osteocalcin, 775/3613 tokens.

Row 6: M A 6, Oldrini et al. 2022, Orthopedic, 12 R C T s, Functional Outcomes D A S H, P R W E, and E Q-5 D at 3 and 12 months, Range of motion flexion, extension, pronation, and supination at 3, 6, and 12 months, Grip Strength percentage of the contralateral side at 3, 6, and 12 months, and Radiological parameters palar tilt, radial inclination, ulnar variance, and step-off immediately post-surgery and at more than 3 months, 1291/3355 tokens.

Navigate back to Table 1.

### Table 2 Long description

The table is divided into three main sections: Statistics results, Quality assessment results, and Study information results. Each section evaluates four approaches: E X T Baseline, E X T plus Self-reflection, Combined E X T, and Customized E X T.

1. Statistics results: Under Customized E X T, Gemini achieves the highest Precision of 0.952 and Recall of 0.760, earning a Recall rank of 1.0. Grok follows with a Recall rank of 2.0 and GPT with 3.0. For other approaches, Recall ranks are represented by diagonal line icons.

2. Quality assessment results: Grok consistently outperforms other models. In the Customized E X T approach, Grok reaches a Precision of 0.953 and a Recall of 0.782, ranking 1.0. GPT ranks 2.0 and Gemini ranks 3.0.

3. Study information results: Grok maintains the lead in the Customized E X T approach with a Precision of 0.887 and Recall of 0.841, ranking 1.0. Gemini ranks 2.0 and GPT ranks 3.0.

Across all categories, the Customized E X T approach generally yields higher Recall values compared to the Baseline and Self-reflection methods. Bolded values indicate the top-performing model for specific metrics within each approach.

Navigate back to Table 2.

### Figure 3 Long description

The horizontal x-axis is labeled Mean Rank with lower is better in brackets, ranging from 0.5 to 5.0. Four methods are listed vertically from top to bottom.

* Customized E X T is at the top with an orange dot at 1.61. Its horizontal line segment extends from approximately 0.9 to 2.3.

* Combined E X T is second with a purple dot at 2.44. Its horizontal line segment extends from approximately 1.7 to 3.2.

* E X T plus Self-reflection is third with a red dot at 2.44. Its horizontal line segment is identical to Combined E X T, extending from 1.7 to 3.2.

* E X T is at the bottom with a blue dot at 3.50. Its horizontal line segment extends from approximately 2.8 to 4.2.

The orange line for Customized E X T overlaps with the purple and red lines but does not overlap with the blue line for E X T, indicating a significant performance difference between Customized E X T and E X T.

Navigate back to Figure 3.

### Table 3 Long description

The table consists of four columns: Model, Method, Delta Recall, and Delta Precision.

For the G P T model:

* E X T plus Self-reflection: Delta Recall 0.011, Delta Precision 0.001.

* Combined E X T: Delta Recall 0.143, Delta Precision 0.076.

* Customized E X T: Delta Recall 0.135, Delta Precision minus 0.023.

For the Gemini model:

* E X T plus Self-reflection: Delta Recall 0.016, Delta Precision 0.001.

* Combined E X T: Delta Recall 0.038, Delta Precision 0.008.

* Customized E X T: Delta Recall 0.172, Delta Precision 0.007.

For the Grok model:

* E X T plus Self-reflection: Delta Recall 0.027, Delta Precision 0.001.

* Combined E X T: Delta Recall minus 0.003, Delta Precision minus 0.023.

* Customized E X T: Delta Recall 0.139, Delta Precision minus 0.009.

Bolded values indicating peak performance include G P T Combined E X T (both metrics), Gemini Combined E X T (Precision) and Customized E X T (Recall), and Grok Self-reflection (Precision) and Customized E X T (Recall).

Navigate back to Table 3.

### Figure 4 Long description

The six panels are organized into three rows. Each row represents a category: Statistics at the top, Quality Assessment in the middle, and Study Information at the bottom. Each row contains two bar charts: Recall on the left and Precision on the right. The y-axis for all charts ranges from 0.0 to 1.0. The x-axis lists six meta-analyses labeled M A 1 through M A 6.

Four methods are compared in each cluster of bars: E X T (blue), E X T plus Self-reflection (orange), Combined E X T (dark green), and Customized E X T (maroon).

* Statistics Row: Recall shows high variability, with Customized E X T reaching above 0.8 in M A 3 but other methods often falling below 0.4. Precision is consistently high across all methods, generally staying between 0.8 and 1.0.

* Quality Assessment Row: Both Recall and Precision show high stability across all six meta-analyses. Most bars cluster between 0.7 and 0.9 for Recall and between 0.8 and 1.0 for Precision, with very little variance between the four methods.

* Study Information Row: Recall shows moderate performance, generally between 0.5 and 0.8, with M A 4 being the lowest performer. Precision remains high and stable, consistently above 0.8 for almost all meta-analyses and methods.

A legend at the bottom center identifies the four color-coded extraction methods.

Navigate back to Figure 4.

### Figure 5 Long description

The figure consists of three rows representing different data categories: Statistics, Quality Assessment, and Study Information. Each row contains two bar charts: Recall on the left and Precision on the right. The y-axis for all charts ranges from 0.0 to 1.0. The x-axis lists six meta-analyses labeled M A 1 through M A 6. Three models are compared in grouped bars: gpt-4o-mini in blue, gemini-2.0-flash in orange, and grok-3 in dark green.

* Statistics Row: Recall shows high variability with values ranging from approximately 0.15 to 0.75. Precision is generally higher, mostly between 0.7 and 0.95.

* Quality Assessment Row: Both Recall and Precision show more stability across M A 1 to M A 6, with most values clustered between 0.6 and 1.0. Grok-3 frequently shows the highest performance in this category.

* Study Information Row: Recall shows a significant dip for M A 4 across all models. Precision remains consistently high, generally above 0.8 for all models across the six meta-analyses.

Overall, grok-3 and gemini-2.0-flash tend to outperform gpt-4o-mini across most metrics and meta-analyses, with grok-3 often reaching the highest peaks in Precision.

Navigate back to Figure 5.

### Table 4 Long description

The table consists of five columns: Dataset, Method, Model, Precision, and Recall. It is divided into three thematic sections.

1. Statistics results:

- M A 1: Customized E X T, G P T, Precision 0.840, Recall 0.792.

- M A 2: Customized E X T, Gemini, Precision 0.947, Recall 0.608.

- M A 3: Customized E X T, Gemini, Precision 0.927, Recall 0.893.

- M A 4: Customized E X T, Grok, Precision 0.970, Recall 0.674.

- M A 5: Customized E X T, Gemini, Precision 0.986, Recall 0.799.

- M A 6: Customized E X T, Gemini, Precision 0.972, Recall 0.785.

2. Quality assessment results:

- M A 1: Customized E X T, Grok, Precision 1.000, Recall 0.684.

- M A 2: Customized E X T, Grok, Precision 0.962, Recall 0.773.

- M A 3: Combined E X T, Combined, Precision 0.871, Recall 0.900.

- M A 4: E X T, Grok, Precision 1.000, Recall 0.778.

- M A 5: Customized E X T, Grok, Precision 0.935, Recall 0.878.

- M A 6: E X T, Grok, Precision 0.955, Recall 0.810.

3. Study information results:

- M A 1: Customized E X T, Grok, Precision 0.826, Recall 0.919.

- M A 2: Customized E X T, Gemini, Precision 0.841, Recall 0.894.

- M A 3: Customized E X T, Gemini, Precision 0.903, Recall 0.829.

- M A 4: E X T plus Self-reflection, Grok, Precision 0.980, Recall 0.639.

- M A 5: Customized E X T, Gemini, Precision 0.926, Recall 0.965.

- M A 6: Customized E X T, Grok, Precision 0.842, Recall 0.882.

Navigate back to Table 4.

### Figure 6 Long description

The diagram consists of a central pyramid divided into three horizontal segments.

At the base is Tier 1, colored blue. To its left, a blue circle indicates 10 percent human effort. To its right, a blue rounded box is labeled Achievable Now. Below this box, a text field states: Fields that can be reliably extracted with current L L M capabilities, requiring little to no human intervention.

The middle section is Tier 2, colored green. To its left, a green circle indicates 20 percent human effort. To its right, a green rounded box is labeled Challenging but Automatable. Below this box, a text field states: Fields that can be automated with targeted prompt design and structured validation.

At the apex is Tier 3, colored orange. To its left, an orange circle indicates 40 percent human effort. To its right, an orange rounded box is labeled Human Judgment Essential. Below this box, a text field states: Tasks that remain fundamentally interpretive or ambiguous and will likely require human involvement.

An upward-pointing arrow runs along the left side of the pyramid, connecting the 10 percent, 20 percent, and 40 percent markers. A note at the top left specifies that the percentage means estimated proportion of human effort.

Navigate back to Figure 6.

### Table 5 Long description

The table consists of four columns: Category, Role and sensitivity, Automation plan (target plus reviewer strategy), and Roadmap tier.

* Row 1: Statistical category. Role is core to meta-analytic conclusions where errors distort estimates. Automation plan targets Recall greater than or equal to 90 percent and Precision greater than or equal to 95 percent using customized prompts with human verification of effect direction. Roadmap tier is Tier 3: Challenging; requires prompt tuning and manual validation.

* Row 2: Quality assessment category. Role supports bias judgments and is semi-subjective. Automation plan targets Recall greater than or equal to 85 percent and Precision greater than or equal to 95 percent using ensemble methods or self-reflection. Roadmap tier is Tier 2: Automation feasible.

* Row 3: Study information category. Role is contextual metadata with high error tolerance. Automation plan targets Recall greater than or equal to 80 percent and Precision greater than or equal to 90 percent using generic prompts with optional review. Roadmap tier is Tier 1: Achievable now with minimal oversight.

Navigate back to Table 5.
