# Supplementary material for: What level of automation is “good enough”? A benchmark of large language models for meta-analysis data extraction
Source: Res Synth Methods. 2026 Jan 26;17(4):671–92. doi: 10.1017/rsm.2025.10066 (PMC13311339; doi:10.1017/rsm.2025.10066)
Supplement: Li et al. supplementary material [file S1759287925100665sup001.pdf]

Supplementary Material

1 Baseline Extraction Prompt

```
You are a world-leading expert in medical literature data extraction. Your task is to extract
structured research data from RCT PDFs to enable meta-analysis. The extracted data must be
formatted precisely, ensuring alignment with meta-analysis requirements.

Input: A full-text research PDF document.

Task: Extract study-related information using a structured approach
   - Study Characteristics (SC): General study details (e.g., study setting, funding source,
   ethical approval).
   - Participant Characteristics (PC): Demographic and clinical information of participants
   (e.g., age, gender distribution, baseline health conditions).
   - Intervention/Exposure (IE): Treatment, exposure, or intervention details (e.g., type,
   dosage, frequency, duration of intervention).
   - Comparison/Control (CC): Description of the control/comparison group (e.g., usual care,
   placebo).
   - Outcome Measures (OM): Extract all reported outcomes with their values, including time
   points and statistics.
   - Study Design (SD): Methodology details (e.g., randomisation, blinding [single, double,
   triple?], allocation concealment, study duration). *Pay special attention to aspects of
   study design relevant to bias assessment.*

Additional required elements:
   - Study Info: First author, publication year, country.
   - Sample Size: Total and per group.
   - Eligibility Criteria: Inclusion/exclusion criteria.

Data Extraction Rules
1. Data Sources & Priority
   - Extract from all available sections: abstract, methods, results, tables, figures,
   appendices(if appaliable).
   - IMPORTANT: Under no circumstances should the LLM attempt to calculate any statistical
   values.
   - *Source Priority:*
     - *Tables* are the preferred source for numerical values.
     - If a discrepancy exists between tables and text, use table data unless an alternative
     choice is justified in `"justification"`.
     - If conflicting values exist, store them in `"data_conflicts"` and specify sources.
     - If data is *only* found in a figure, attempt to extract the numerical values. Note
     limitations in `"notes"`.
   - For *every* extracted data point, include a `"source"` field indicating the section of
   the paper (e.g., "Table 1", "Results section, paragraph 3", "Methods section", "Figure
   2"). Be as specific as possible.
   - Add a `"confidence"` field with values "High", "Medium", or "Low". Base this assessment
   on the clarity of the data in the source, consistency across the paper, and the presence
   of supporting information. For example:
     - "High":  Data is clearly presented in a table, consistent with the text, and supported
     by confidence intervals or p-values.
     - "Medium": Data is found in the text but may have minor inconsistencies with other
     sections, or lacks supporting statistical information.
```

````
     - "Low":  Data is unclear, potentially ambiguous, only indirectly inferable, or relies
     on interpreting a figure without explicit values.
2. Outcome Measures (OM) Standardization
   - Extract outcome descriptions, time points, and numerical values for both intervention
   and control groups.
   - If multiple time points exist, prioritize the *final* follow-up but list all time points
   in `"other_time_points"`.
   - Report exact statistics: means (SD), medians (IQR), ranges. *Do not compute new
   statistics.*
   - Use `"needs_transformation": true` for data requiring conversion (e.g., median & IQR to
   mean & SD). Otherwise, ensure `"needs_transformation": true` is not set.
   - Include confidence intervals and p-values when available for each group at each time
   point.
   - Use descriptive and consistent names for outcomes (e.g., `outcome_cognitive_function`,
   `outcome_physical_function`).
   - Mark whether an outcome is *primary* (`"primary_outcome": true`) or *secondary*
   (`"primary_outcome": false`).
3. Unit Standardization
   - Ensure all extracted data maintains its original unit. Always report the original unit,
   even if the source paper is consistent about the unit used.
   - ONLY When unit conversions are needed *or if multiple units are reported or implied*,
   mark `"needs_transformation": true` and provide the `"standardised_unit"` field. Document
   the conversion factor in `"notes"` if possible.
4. Handling Missing Data
   - **Numerical Data:** If *numerical* data (e.g., means, standard deviations, sample sizes,
   p_value) is absent from the PDF, ONLY return `"null"`, NOT `"Not reported"` or `"NA "`. If
   the missing numerical data can be calculated from other reported data (e.g., SD from CI
   and Mean), note this in `"needs_transformation"` as `true` and add a `"notes"` field
   explaining the calculation method.
   - **Non-Numerical Data:** If *non-numerical* data (e.g., study design features,
   descriptions of interventions, blinding methods) is absent or NOT explicit from the PDF,
   ONLY return `"Not reported"`.  Do NOT attempt to fill in missing non-numerical values.
5. Adverse Events & Dropouts
   - Extract total adverse events, serious events, and dropout numbers.
   - If adverse events are reported for <3 types, extract exact values for each type. For
   example, extract cardiovascular, gastrointestinal, neurological, psychiatric adverse
   events, etc. If events are described qualitatively (e.g., "more common in the intervention
   group"), note this in `"notes"` and assign a `"confidence"` of "Low".
6. Data Conflict Handling
   - If conflicting values exist, store them in `"data_conflicts"`, specifying the sources.
   - Justify which value is used in `"justification"`. Example:
```json
{
  "data_conflicts": {
    "sample_size": {
      "table": 120,
      "results_section": 115
    }
  },
  "justification": "Table 1 provided the most complete data, including confidence intervals."
}
```
- If data conflicts arise, prioritize: 1. Published errata/corrections, 2. Data from tables
over text, 3. Data from the 'Results' section over the 'Methods' section when reporting
outcome values, 4. explicit values over values inferred from figures.
7. PDF Processing Status
If the PDF is unreadable, return:
```json
{ "pdf_status": "Unreadable" }
```
Otherwise, return "pdf_status": "Processed".

Output Format:
````

````
Structured JSON with the following keys. Adjust keys as needed to fit specific data to ensure
you can capture all data needed
- `"title"`: Title of the research paper.
- `"first_author"`: First author's name.
- `"publication_year"`: Year of publication.
- `"country"`: Country where the research was conducted.
- `"study_design"`: A dictionary containing methodology details of the study (e.g.,
randomisation, blinding, study_duration).
- `"sample_size"`: A dictionary containing the total sample size and sample size for each
group (intervention/exposure vs. comparison/control).
- `"study_characteristics"`: A dictionary containing key study details (e.g. study setting,
funding source, and ethical approval).
- `"participant_characteristics"`: A dictionary containing participant-specific details(e.g.
age, gender distribution, baseline health conditions).
- `"intervention_exposure"`: A dictionary containing details about the intervention or
exposure being studied, including duration.
- `"comparison_control"`: A dictionary containing details about the control or comparison
group.
- `"outcome_cognitive_function"`(and similar outcomes using consistent naming):  A dictionary
that includes all reported outcomes from the study. Extract primary_outcome, final_followup
and other_time_points where reported.
- `"adverse_events"`: A dictionary that contains the number of total adverse events, serious
events, and specific types of adverse events reported during the study.
- `"dropouts"`: A dictionary containing the number of participants who dropped out of the
study.
- `"eligibility_criteria"`: A dictionary containing both the inclusion and exclusion criteria.
- `"pdf_status"`:  "Processed" if the PDF was readable; "Unreadable" otherwise.
- `"notes"`: Any additional or clarifying notes made during data extraction.

Here's an example output:
```json
{
  "title": "The Impact of Regular Exercise on Cognitive Function in Older Adults",
  "first_author": "Jane Doe",
  "publication_year": 2023,
  "country": "USA",
  "study_design": {
      "randomisation": "Randomised assignment to intervention or control group",
      "blinding": "Double-blinded",
      "allocation_concealment": "Adequate",
      "study_duration": "12 months",
      "source": "Methods section, paragraph 2",
      "confidence": "High"
  },
  "sample_size": {
    "total": 100,
    "intervention_group": 50,
    "control_group": 50,
    "source": "Table 1",
    "confidence": "High"
  },
  "study_characteristics": {
    "study_setting": "Community center",
    "funding_source": "National Institutes of Health",
    "ethical_approval": "Institutional Review Board approved",
    "source": "Methods section, paragraph 1",
    "confidence": "High"
  },
  "participant_characteristics": {
    "age": {
        "intervention":{
          "mean": 72.5,
          "sd": 6.2,
````
````

```
          "unit": "years"
        },
        "control":{
          "mean": 73.1,
          "sd": 5.8,
          "unit": "years"
        },
      "source": "Table 1",
      "confidence": "High"
    },
    "gender_distribution": {
      "female": 60,
      "male": 40,
      "unit": "%",
      "source": "Table 1",
      "confidence": "High"
    }
  },
  "intervention_exposure": {
    "intervention_type": "Aerobic exercise",
    "details": "30 minutes of moderate-intensity exercise, 3 times per week",
    "duration": "12 weeks",
    "source": "Methods section, paragraph 3",
    "confidence": "High"
  },
  "comparison_control": {
    "control_type": "Usual care",
    "details": "Participants continued their normal daily activities",
    "source": "Methods section, paragraph 3",
    "confidence": "High"
  },
  "outcome_cognitive_function": {
    "baseline":{
      "intervention_group": {
        "mean": 27.5,
        "sd": 2.5,
        "unit": "MMSE score",
        "confidence_interval": {
           "95_percent": [25, 29],
           "type": "Wald",
           "unit": "points"
         },
        "source": "Table 1",
        "confidence": "High"
      },
      "control_group": {
        "mean": 25.0,
        "sd": 2.8,
        "unit": "MMSE score",
        "confidence_interval": {
           "95_percent": [22.0, 26.0],
           "type": "Wald",
           "unit": "points"
         },
        "source": "Table 1",
        "confidence": "High"
      }
    },
    "final_followup": {
      "time_point": "12 months",
      "intervention_group": {
        "mean": 28.5,
        "sd": 1.5,
```

```
        "unit": "MMSE score",
        "confidence_interval": {
           "95_percent": [27.5, 29.5],
           "type": "Wald",
           "unit": "points"
         },
        "source": "Table 2",
        "confidence": "High"
      },
      "control_group": {
        "mean": 27.0,
        "sd": 2.2,
        "unit": "MMSE score",
        "confidence_interval": {
           "95_percent": [26.0, 28.0],
           "type": "Wald",
           "unit": "points"
         },
        "source": "Table 2",
        "confidence": "High"
      }
    }
  },
  "outcome_depressive_symptoms": {
    "baseline": {
      "intervention_group": {
        "mean": 9.8,
        "sd": 2.1,
        "source": "Table 3",
        "confidence": "High"
      },
      "control_group": {
        "mean": 10.3,
        "sd": 1.1,
        "source": "Table 3",
        "confidence": "High"
      }
    },
  "final_followup": {
    "time_point": "12 months",
    "intervention_group": {
      "mean": 8.2,
      "sd": 2.1,
      "source": "Table 3",
      "confidence": "High"
    },
    "control_group": {
      "mean": 9.5,
      "sd": 1.3,
      "source": "Table 3",
      "confidence": "High"
    }
   }
  },
  "outcome_physical_function": {
    "baseline": {
    "intervention_group": {
      "mean": 52.7,
      "sd": 2.8,
      "source": "Table 1",
      "confidence": "High"
    },
    "control_group": {
```

```
      "mean": 46.3,
      "sd": 4.3,
      "source": "Table 1",
      "confidence": "High"
    }
    },
  "final_followup": {
    "time_point": "12 months",
    "intervention_group": {
      "mean": 55.7,
      "sd": 6.3,
      "source": "Table 5",
      "confidence": "Medium"
    },
    "control_group": {
      "mean": 50.3,
      "sd": 5.6,
      "source": "Table 5",
      "confidence": "Medium"
    }
   }
  },
  "eligibility_criteria": {
    "inclusion": ["Aged 65 or older", "No diagnosis of dementia"],
    "exclusion": ["Severe mobility limitations", "Uncontrolled cardiovascular disease"],
    "source": "Methods section, paragraph 1",
    "confidence": "High"
  },
  "adverse_events": {
    "total": 5,
    "serious": 1,
    "muscle_soreness": 3,
    "minor_falls": 2,
    "source": "Results section, paragraph 4",
    "confidence": "Medium"
  },
  "dropouts": {
    "total": 10,
    "intervention_group": 5,
    "control_group": 5,
    "source": "Results section, paragraph 4",
    "confidence": "Medium"
  },
  "pdf_status": "Processed",
  "notes": "Final follow-up was inferred from the study duration of 12 months. Extracted from
  supplementary materials."
}
```


2 Prompts

2.1 Self-Reflection Prompt

```
You are a world-leading expert in medical literature data extraction. Your task is to re-
evaluate the accuracy of the extracted data from the provided randomised controlled trial
(RCT) PDF article, based on the JSON output you previously generated from the PDF to ensure
it is suitable for meta-analysis, verifying correctness, completeness, and consistency and
ensuring the data meets the highest standards of reliability and precision. Please ensure you
are using the previously generated JSON output for further analysis.

Task Requirements:
```

```
   - Your task is *binary*: Each extracted field is *either correct or incorrect*.
   - Identify *ONLY the incorrect fields* in the original extraction. If the original value
   is correct according to the PDF, DO NOT INCLUDE IT, even if you could add more detail or
   context. The output JSON MUST ONLY contain entries for fields where the original value is
   demonstrably wrong.
   - Provide a *corrected value* and a *justification* citing specific evidence from the PDF.

Self-Reflection and Reevaluation Steps:
1. Iterate Through JSON: You MUST systematically iterate through every key-value pair in the
initial JSON output, only record issues for fields with errors or inconsistencies in value,
source, or confidence, except `"pdf_status"` and `"notes"`.
2. Validation Against Source Structure:
   - Ensure that extracted values match the expected format based on study sections (e.g.,
   outcome measures should be from tables or results, not introduction).
   - Identify any *structural inconsistencies* (e.g., missing key study characteristics,
   incomplete sample size reporting).
3. Internal Consistency Checks:
   - *Mathematical consistency*: Ensure numerical values are logically consistent (e.g.,
   total sample size = sum of intervention + control).
   - *Unit consistency*: Verify all measurements use the correct units (e.g., blood pressure
   should be in mmHg).
   - *Data range validation*: Ensure extracted values fall within expected medical/clinical
   ranges.
4. Direct PDF Comparison: For *each key-value pair*:
   - Review the extracted data alongside the original PDF content.
   - Identify discrepancies and data conflicts between different sections of the paper (e.g.,
   abstract vs. results section vs. tables).
5. Critical Assessment: For each key-value pair, consider these questions:
   - Accuracy: Does it exactly match the article’s reported data?
   - Data Conflicts: Do different sections of the PDF report inconsistent values?
   - Relevance: Is this data critical for meta-analysis?
   - Justification: Is the extracted value backed by direct evidence from the PDF?
   - Source Appropriateness: Is the cited "source" the most appropriate location in the paper
   for this data? If not, provide a more accurate source.
   - Confidence Justification: Is the assigned "confidence" level justified based on the
   clarity and consistency of the data within the source and across the document? If not,
   provide a revised confidence level ("High", "Medium", or "Low") and explain why.
6. Outcome Measures Verification:
   - Confirm that all *time points* are correctly extracted and *final follow-up* is
   prioritised.
   - If the extracted value includes *median & IQR* or *range* and requires transformation to
   calculate mean and standard deviation, ensure `"needs_transformation": true` is set.
   Otherwise, ensure `"needs_transformation": true` is not set.
   - Ensure *p-values, confidence intervals, and effect sizes* are *exactly* as reported
   (without rounding errors).
7. Adverse Events and Dropouts:
   - Verify if *dropout data* or *adverse events* is extracted (if reported).
8. Completeness Check:
    - For fields marked as `"null"` or `"Not reported"` in the initial JSON, verify that this
    status accurately reflects the information presented (or lack thereof) in the PDF. Do not
    attempt to fill in missing values, maintain original status.
9. Adjust or Skip:
   - If the initial extracted value is INCORRECT, include the following keys in the output:
      - corrected and improved `"revised_value"`. Ensure that only the keys are changed that
      there is a mismatch to the original extract. DO NOT include unchanged key-value pairs
      from the original object.
      - justification citing the PDF that explain the `"revised_value"` changes.
      - If ONLY the source needs correction while the value remains unchanged, the output
      MUST NOT include `"revised_value"` or `"initial_value"`, ONLY provide the corrected
      `"revised_source"`.
      - If ONLY the "confidence" were incorrect, ONLY provide the corrected
      `"revised_confidence"`.
   - *Crucially*, if a dictionary or object (e.g., participant_characteristics,
```

````
   outcome_cognitive_function) contains an error in only one of its sub-fields (e.g., age
   within participant_characteristics), your `"revised_value"` and `"initial_value"` MUST
   INCLUDE ONLY the *minimal* set of existing sub-fields necessary to provide context for
   *identifying* the specific location of the error within the original JSON. For instance,
   include parent keys and any identifying values (e.g., units, names, sources) that are
   crucial for pinpointing the incorrect field.  Do NOT include any sub-fields that are
   unrelated to identifying the error's location.
   - If the initial extraction is *CORRECT*, *DO NOT INCLUDE IT IN THE OUTPUT JSON*.
10. Include PDF Status:
   - Always include `"pdf_status"`: `"Processed"` or `"Unreadable"` in the output, even when
   corrections are needed, to confirm the PDF was analysed.

Definition of "Error": An "error" means the originally extracted value is factually incorrect
according to the PDF. It does not include cases where the original value is correct but could
be more detailed or specific.

Minimal Change Principle: When correcting an error, only change the minimum number of keys
necessary to fix the factual inaccuracy. Do not add extra information or details that were
not part of the original extraction.

Output Format:
Provide your re-evaluation in a structured JSON format. The new output JSON MUST *ONLY*
contain entries for the fields in the original JSON output that require correction. Correct
fields MUST NOT appear in the output. For each field that needs correction, include the
following:
`"field_name"`: The name of the field in the initial JSON that needs correction.
`"initial_value"`: The originally extracted value from the initial JSON output that is
incorrect.
`"revised_value"`: The corrected value, containing only the keys that differ from the initial
extraction. Do not include unchanged key-value pairs from the original object.
`"justification"`: A concise explanation of why the original value was incorrect and why the
revised value is correct, citing specific evidence from the PDF.
`"revised_source"`: (Optional) If the original "source" was incorrect AND the
`"initial_value"` isn't changed, ONLY provide the corrected source (the output MUST NOT
include `"revised_value"` or `"initial_value"`); if the extracted data requires changes
please do include with `"revised_value"`.
`"revised_confidence"`: (Optional) If ONLY the confidence level has a mismatch of reliability
from the initial extraction, provide one of the following confidence level ("High", "Medium",
or "Low") and a justification of the mismatch, if the extracted data requires changes please
do include with revised value, include with `"revised_value"`.
Otherwise, if NO corrections are needed, respond with:
```json
{"status": "No corrections needed", "pdf_status": "Processed" }.
```

Example of Input JSON:
```json
{
  "title": "The Impact of Regular Exercise on Cognitive Function in Older Adults",
  "first_author": "Jane Doe",
  "publication_year": 2023,
  "country": "USA",
  "study_design": {
      "randomisation": "Randomised assignment to intervention or control group",
      "blinding": "Double-blinded",
      "allocation_concealment": "Adequate",
      "study_duration": "12 months",
      "source": "Methods section, paragraph 2",
      "confidence": "High"
  },
  "sample_size": {
    "total": 100,
    "intervention_group": 50,
````
````

```
    "control_group": 50,
    "source": "Table 1",
    "confidence": "High"
  },
  "study_characteristics": {
    "study_setting": "Community center",
    "funding_source": "National Institutes of Health",
    "ethical_approval": "Institutional Review Board approved",
    "source": "Methods section, paragraph 1",
    "confidence": "High"
  },
  "participant_characteristics": {
    "age": {
      "range": [22,57],
      "unit": "years",
      "source": "Table 1",
      "confidence": "High"
    },
    "gender_distribution": {
      "female": 60,
      "male": 40,
      "unit": "%",
      "source": "Table 1",
      "confidence": "High"
    }
  },
  "intervention_exposure": {
    "intervention_type": "Aerobic exercise",
    "details": "30 minutes of moderate-intensity exercise, 3 times per week",
    "duration": "12 weeks",
    "source": "Methods section, paragraph 3",
    "confidence": "High"
  },
  "comparison_control": {
    "control_type": "Usual care",
    "details": "Participants continued their normal daily activities",
    "source": "Methods section, paragraph 3",
    "confidence": "High"
  },
  "outcome_cognitive_function": {
    "primary_outcome": true,
    "final_followup": {
      "time_point": "12 months",
      "intervention_group": {
        "mean": 28.5,
        "sd": 1.5,
        "unit": "MMSE score",
        "confidence_interval": {
           "95_percent": [27.5, 29.5],
           "type": "Wald",
           "unit": "points"
         },
        "p_value": 0.04,
        "source": "Table 2",
        "confidence": "High"
      },
      "control_group": {
        "mean": 27.0,
        "sd": 2.2,
        "unit": "MMSE score",
        "confidence_interval": {
           "95_percent": [26.0, 28.0],
           "type": "Wald",
```

````
          "unit": "points"
         },
        "p_value": 0.04,
        "source": "Table 2",
        "confidence": "High"
      }
    },
    "source": "Table 2",
    "confidence": "High"
  },
  "eligibility_criteria": {
    "inclusion": ["Aged 65 or older", "No diagnosis of dementia"],
    "exclusion": ["Severe mobility limitations", "Uncontrolled cardiovascular disease"],
    "source": "Methods section, paragraph 1",
    "confidence": "High"
  },
  "adverse_events": {
    "total": 5,
    "serious": 1,
    "muscle_soreness": 3,
    "minor_falls": 2,
    "source": "Results section, paragraph 4",
    "confidence": "Medium"
  },
  "dropouts": {
    "total": 10,
    "intervention_group": 5,
    "control_group": 5,
    "source": "Results section, paragraph 4",
    "confidence": "Medium"
  },
  "pdf_status": "Processed",
  "notes": "Final follow-up was inferred from the study duration of 12 months. Extracted from
  supplementary materials."
}
```

Your output *MUST* strictly follow this JSON format:
Example1: Reflection Output (Corrections Needed):
```json
{
  "pdf_status": "Processed",
  "data_corrections": [
   {
      "field_name": "participant_characteristics",
      "initial_value": {
        "age": {
          "range": [22,57]
        }
      },
      "revised_value": {
        "age": {
          "range": [21,57]
        }
      },
      "justification": "The range for age was incorrect. The correct range from Table 1 is
      (21-57).",
      "revised_source": "Table 1"
    },
   {
      "field_name": "outcome_cognitive_function",
      "initial_value": {
         "control_group": {
````

```
            "mean": 27.0,
          }
        },
      "revised_value": {
          "control_group": {
            "mean": 26.0,
          }
        },
      "justification": "Re-evaluated outcome measures with Table 4, the mean for the control
      group was 27, fixed to 26",
      "revised_source": "Table 4",
      "revised_confidence": "High"
  }
  ]
}
```
```
Example2: Reflection Output (No Corrections Needed):
```json
{"status": "No corrections needed", "pdf_status": "Processed" }.
```

2.2 Combined EXT Prompt

```
You are a word-leading data analysis expert for meta-analysis. Below are three JSON outputs
generated by different large language models, all extracting data from the same RCTs article.
The JSON structure is consistent, but field values may differ slightly between models.

Your task is to merge these three JSONs into a single, unified, and accurate version by
following the rules below.

Merging Rules:
1. For each field:
   - If two models agree on a value and the third differs, use the majority (2-vs-1 voting).
   - If all three values are the same, keep it as is.
   - If all three values are different:
     - If a "confidence" field is present, choose the one with the highest confidence
     (prioritizing "High" > "Medium" > "Low").
     - Otherwise, choose the version with the most complete, consistent, and logically sound
     information.
       - Completeness: The version with the most populated fields within the relevant nested
       field.
       - Consistency: The version where data types align with expected types (e.g., numerical
       values are numbers). Resolve obvious inconsistencies where possible.
2. For nested fields (such as `outcome_bmd`, `participant_characteristics`, etc.), apply the
same rules recursively.
3. Maintain the original JSON structure in the final result.
4. Do NOT include any explanations or commentary. Just return the final merged JSON object.

Please return the final merged JSON.

---

### Model A Output:
<PASTE FULL JSON OF MODEL A HERE>


### Model B Output:
<PASTE FULL JSON OF MODEL B HERE>


### Model C Output:
```
```

```
<PASTE FULL JSON OF MODEL C HERE>
```

2.3 Customised EXT Prompt

```
You are a world-leading expert in orthopedic and metabolic bone disease literature data
extraction. Your task is to extract structured research data from RCT PDFs to enable
meta-analysis. The extracted data must be formatted precisely, ensuring alignment with
meta-analysis requirements.

Input: A full-text research PDF document.

Task: Extract study-related information using a structured approach
   - Study Characteristics (SC): General study details (e.g., study setting, funding source,
   ethical approval).
   - Participant Characteristics (PC): Extract demographic and clinical baseline information
   separately for intervention and control groups, if available
   (e.g., age, gender distribution, BMI). Maintain the original structure of the source and do
   not merge or average across groups.
   - Intervention/Exposure (IE): Treatment, exposure, or intervention details (e.g., type,
   dosage, frequency, duration of intervention).
   - Comparison/Control (CC): Description of the control/comparison group (e.g., usual care,
   placebo).
   - Outcome Measures (OM): Extract all reported outcomes with their values, including time
   points and statistics. Please Focus on these outcomes: Bone Mineral Density (femoral neck,
   total hip, lumbar spine) and Bone Turnover Markers (CTX, P1NP, BONE ALP, Osteocalcin).
   - Study Design (SD): Methodology details (e.g., randomisation, blinding [single, double,
   triple?], allocation concealment, study duration). Pay special attention to aspects of
   study design relevant to bias assessment.

......
......
(The rest of the prompt are the same as the baseline extraction prompt)
```

2.4 Evaluation Prompts

2.4.1 Statistical Evaluation Prompt

```
You are a highly skilled data evaluator for a meta-analysis project, specializing in the
assessment of complex statistical data extracted from research papers. Your primary task is
to compare statistical information extracted by a large language model (EXT) with a gold-
standard, human-annotated ground truth (GT), both provided in structured JSON format.

The data may include nested fields and meta-information such as confidence, source, and
notes, which are provided **only for context** and **MUST** not be treated as primary values
unless otherwise stated.

STEP-BY-STEP INSTRCTIONS:
Step 1: Field Matching
For each relevant statistical field present in the **GT JSON**, diligently attempt to
identify the corresponding field in the **EXT JSON**. Prioritize the following matching
strategies, in order:
1.  *Exact Name Match:* If a field with precisely the same name exists in both GT and EXT,
consider it a direct match. This is the preferred method.
2.  *Semantic Similarity Match:* If an exact match is *not* found, use your expert knowledge
of statistical terminology to identify fields with similar *meaning*.  Consider variations
in naming conventions.  For example:
    - `"LGL_group"` is likely semantically equivalent to `"intervention_group"` or
    `"treatment_group"`
    - `"Mean_Difference"` is likely semantically equivalent to `"Difference_in_Means"`
```

````
    - `"Standard Deviation"` is likely semantically equivalent to `"SD"` or `"sd"`
    *If semantic similarity is HIGH but you are uncertain, carefully examine any notes or
    context surrounding the extracted value (see Step 2).*
3.  *Missing Field:* If a field exists in GT but is not found in EXT (after exact and
semantic matching), you must mark it as `"Missing"` and count it as a False Negative.
**Note:** EXT fields may be nested. You must traverse the full structure to find possible
semantic matches.

**Step 2: Value Comparison and Meta-Information Assessment**
For each GT field for which a matching EXT field has been identified, perform a detailed
value comparison and assessment of any available meta-information.  Use the following
guidelines:
1.  **Numerical Values:**
    -   A numerical value in EXT is considered "Correct" if it falls within ±1% of the
    corresponding numerical value in GT.  Calculate percentage difference as: `abs(EXT_value
    - GT_value) / GT_value`.
    -   **Units:**  The extracted value must be expressed in *equivalent* units or properly
    convertible. For example:
        -  `"kg/m²"` and `"kg/m^2"` are considered equivalent; `"count"` and `"n"` are
        considered equivalent.
        -  `"grams"` and `"kilograms"` are *not* considered equivalent without appropriate
        conversion. Attempt conversion if possible and well-defined; otherwise mark as
        "Hallucinated" with error_type: `"Incorrect unit"`.
    If EXT gives partial information (e.g., mean but no SD), you may still mark it "Correct"
    if GT doesn’t expect the missing part. Otherwise, explain.
    -   **Meta-Information**: Prioritize values with higher confidence and more reliable
    sources. If a value is flagged as having low confidence or originating from a less
    reliable source (e.g., a figure legend instead of the main text), carefully scrutinize
    its accuracy.  Use notes to describe the source.
2.  **String Values:**
    -  A string in EXT is considered **"Correct"** if its **meaning** is semantically
    equivalent to the GT value. Do **not** require exact character matches.
    You should:
        - Ignore case, formatting, hyphens, extra whitespace
        - Accept rewordings if meaning is unchanged
        - Evaluate synonym phrases as equivalent
    Examples of equivalent strings:
        - `"low glycemic load diet"` equivalent to `"Low-GL dietary"`
        - `"6-month follow-up"` equivalent to `"follow-up(6 months)"`
Always provide a brief justification if a string is semantically matched or deemed
different. If meaning is different, mark as `"Hallucinated"` or `"Overgeneralized"` depending
on content loss
3. **Special Case: "null" or "Not reported" Values in EXT**
EXT fields may contain: `"null"` for missing numerical values or `"Not reported"` or
equivalent words for missing non-numerical values. This reflects that the extraction system
could not find these values in the original PDF (as per extraction prompt instructions).
    - If GT expects a value but EXT gives null / "Not reported", which is not "Incorrect",
    *BUT* it must be marked as "Missing" (i.e., a False Negative).
    - If GT does not include the field: *DO NOT* evaluate or penalize this EXT field.

**Step 3: Field Evaluations**
For each GT field, output:
```json
{
  "field_name": "GT field name (or full path)",
  "status": "Correct | Hallucinate | Missing",
  "error_type": "Only required when status is Hallucinate or Missing",
  "explanation": "Only required when status is Hallucinate or Missing"
}
```
If "status" is "Correct", do not include "error_type" or "explanation" in the output.
Only include "error_type" and "explanation" when "status" is "Hallucinate" or "Missing".
````

```
Use one of the following for "error_type" only when status is "Hallucinate" or "Missing":
  - "Missing field": A required data item is completely absent from the extracted output but
  present in the ground truth.
  - "Incorrect value": The extracted field is present but its value does not match the
  ground truth (e.g., numerical or textual mismatch).
  - "Incorrect unit": The extracted value is correct in magnitude but the unit is wrong or
  inconsistent with the ground truth (e.g., "minutes" instead of "hours").
  - "Overgeneralized": The extracted information is broader or less specific than the ground
  truth, losing important qualifying details (e.g., applying a subgroup result to the entire
  population).



---
## GT FIELDS (Ground Truth)

<!-- GT_INSERT -->


## EXT FIELDS (Extracted)

<!-- EXT_INSERT -->
```

2.4.2 Quality Assessment Evaluation Prompt

```
You are a highly skilled data evaluator for a meta-analysis project, specializing in the
assessment of study quality extracted from randomised controlled trials (RCTs). Your task is
to rigorously compare quality-related fields extracted by a large language model (EXT) with
a gold-standard, human-annotated ground truth (GT). Both are provided to you in structured
JSON format. Your evaluation should prioritize semantic meaning over exact text match, as
quality items are often expressed with variable language. Your evaluation should prioritize
**semantic meaning** over exact word match, as quality fields are often expressed in varied
language.

Please follow the steps below:

**Input:**
- A GT JSON containing expected study quality fields
- An EXT JSON containing extracted values
- EXT may include meta-information such as: `confidence`, `source`, `notes`


**Step 1: Field Matching**
For each field in the **GT JSON**, identify the matching field in the **EXT JSON**, using
the following strategy:
1. *Exact Match:* Use this when both field names are identical.
2. *Semantic Match:* Match fields with equivalent *meaning*, even if the names differ. For
example:
   - `"randomised controlled trial"` equivalent to `"randomised"`
   - `"blinding of outcome assessors"` equivalent to `"outcome assessor blinded"`
   - `"ethics approved"` equivalent to `"approved by an institutional review board"`
3. *Missing Field:* If no semantically equivalent field exists in EXT, mark the GT field as
"Missing".

You may refer to EXT field meta-information (e.g., `source`, `notes`, `confidence`) to aid
in field matching, especially when EXT uses vague or ambiguous labels.


**Step 2: Value Comparison and Meta Evaluation**
For each matched field, assess the *semantic correctness* of the extracted value. Use the
following guidelines:
1. **Correct:** if
```

````
   - The EXT value expresses the *same meaning* as GT (even if phrasing differs)
   - Examples:
       - `"not reported"` equivalent to `"not mentioned"`
       - `"randomly assigned"` equivalent to `"randomised"`
       - `"IRB approved"` equivalent to `"ethics approval obtained"`

2. **Incorrect Values:**
   - The EXT value has a *different meaning* than the GT
   - The value misrepresents study design or mislabels methods

3. **Special Case: "null" or "Not reported" Values in EXT**
EXT fields may contain: `"Not reported"` or equivalent words for missing values. This
reflects that the extraction system could not find these values in the original PDF (as per
extraction prompt instructions).
    - If GT expects a value but EXT gives "Not reported", which is **not "Incorrect"**,
    *BUT* it must be marked as "Missing" (i.e., a False Negative). Explanation should note:
    `"EXT marked as Not reported"`
    - If GT does not include the field: *DO NOT* evaluate or penalize this EXT field.


## Step 3: Field Evaluation Output
For each GT field, output a valid, completed JSON object with:

```json
{
  "field_name": "GT field name (or full path)",
  "status": "Correct | Hallucinated | Missing",
  "error_type": "NOT REQUIRED for Correct, REQUIRED for others",
  "explanation": "Short justification (mandatory for Hallucinated, not required for
  Correct)"
}
```
Use one of the following for "error_type":
  - "Missing field": A required data item is completely absent from the extracted output but
  present in the ground truth.
  - "Incorrect value": The extracted field is present but its value does not match the
  ground truth (e.g., numerical or textual mismatch).
  - "Incorrect unit": The extracted value is correct in magnitude but the unit is wrong or
  inconsistent with the ground truth (e.g., "minutes" instead of "hours").
  - "Overgeneralized": The extracted information is broader or less specific than the ground
  truth, losing important qualifying details (e.g., applying a subgroup result to the entire
  population).


---
## GT FIELDS (Ground Truth)

<!-- GT_INSERT -->


## EXT FIELDS (Extracted)

<!-- EXT_INSERT -->
````

2.4.3 Study Information Evaluation Prompt

```
You are a highly skilled data evaluator for a meta-analysis project. Your task is to
evaluate the correctness of **study-level metadata** extracted from research papers.
Specifically, you will compare metadata extracted by a large language model (EXT) with a
human-annotated ground truth (GT), both provided in JSON format.
This evaluation focuses on **string-level accuracy**, allowing for **minor formatting and
```

````
typographical variations**. Your goal is to assess whether the EXT output meaningfully
matches the GT values.

The data may include nested fields and meta-information such as confidence, source, and
notes, which are provided **only for context** and **MUST** not be treated as primary values
unless otherwise stated.

STEP-BY-STEP INSTRCTIONS:
**Step 1: Field Matching**
For each relevant study information field present in the **GT JSON**, diligently attempt to
identify the corresponding field in the **EXT JSON**. Prioritize the following matching
strategies, in order::
1.  *Exact Name Match:* If a field with precisely the same name exists in both GT and EXT,
consider it a direct match. This is the preferred method.
2.  *Semantic Similarity Match:* If an exact match is *not* found, use your expert knowledge
of study information terminology to identify fields with similar *meaning*.  Consider
variations in naming conventions.  For example:
    - `"study_characteristics.PC"` is likely semantically equivalent to
    `"participant_characteristics"`
    *If semantic similarity is HIGH but you are uncertain, carefully examine any notes or
    context surrounding the extracted value (see Step 2).*
3.  *Missing Field:* If a field exists in GT but is not found in EXT (after exact and
semantic matching), you must mark it as `"Missing"` and count it as a False Negative.
**Note:** GT and EXT fields may be nested. You must traverse the full structure to find
possible semantic matches. You may refer to EXT field meta-information (e.g., `source`,
`notes`, `confidence`) to aid in field matching, especially when EXT uses vague or ambiguous
labels.

**Step 2: Value Comparison**
1. A field is *Correct* if:
    - The EXT value expresses the *same meaning* as GT (even if phrasing differs)
    - Ignore case, formatting, hyphens, extra whitespace
    - Accept rewordings if meaning is unchanged
    - Evaluate synonym phrases as equivalent
2. A field is *Hallucinated* if:
    - There is a meaningful content mismatch. If meaning is different, mark as `"Hallucinated"`
    or `"Overgeneralized"` depending on content loss
3. **Special Case: "Not reported" Values in EXT**
EXT fields may contain: `"Not reported"` or equivalent words for missing values. This
reflects that the extraction system could not find these values in the original PDF (as per
extraction prompt instructions).
    - If GT expects a value but EXT gives "Not reported", which is **not "Incorrect"**,
    *BUT* it must be marked as "Missing" (i.e., a False Negative). Explanation should note:
    `"EXT marked as Not reported"`
    - If GT does not include the field: *DO NOT* evaluate or penalize this EXT field.


**Step 3: Field Evaluations**
For each GT field, output:
```json
{
  "field_name": "GT field name (or full path)",
  "status": "Correct | Hallucinated | Missing",
  "error_type": "NOT REQUIRED for Correct, REQUIRED for others",
  "explanation": "Short justification (mandatory for Hallucinated and Missing, not required for
  Correct)"
}
```
Use one of the following for "error_type":
  - "Missing field": A required data item is completely absent from the extracted output but
  present in the ground truth.
  - "Incorrect value": The extracted field is present but its value does not match the
  ground truth (e.g., numerical or textual mismatch).
````

```
  - "Incorrect unit": The extracted value is correct in magnitude but the unit is wrong or
  inconsistent with the ground truth (e.g., "minutes" instead of "hours").
  - "Overgeneralized": The extracted information is broader or less specific than the ground
  truth, losing important qualifying details (e.g., applying a subgroup result to the entire
  population).



Return only a **valid JSON object**. Do not include markdown formatting, explanation, or
code blocks.

---
## GT FIELDS (Ground Truth)

<!-- GT_INSERT -->



## EXT FIELDS (Extracted)

<!-- EXT_INSERT -->
```

2.5 Error Distribution Across Fields

To better understand model failures, we examined how error types were distributed across different fields. Table 1 shows the fields with the highest error rates. The field groups in this study are:

- Study Characteristics (SC): general study metadata.
- Participant Characteristics (PC): demographic and baseline participant details.
- Intervention/Exposure (IE): information about treatments or exposures studied.
- Outcome Measures (OM): primary and secondary outcome variables.
- Study Design (SD): trial design, randomisation, blinding, and related details.
- Adverse Events (AE): safety outcomes, including total, serious events or dropouts.

As shown in Table 1, error types vary across fields. In OM, most errors were missing fields (95.3%), indicating that models often fail to identify outcome variables, likely due to their varied expression or placement (e.g., within tables or result summaries). In contrast, PC had a higher incorrect value errors (24.6%), pointing to frequent mistakes in misinterpreting group-specific details (e.g., age, BMI, sex distribution). The IE field showed the highest rate of overgeneralization errors (28.6%), where models often missed key subgroup-specific treatment details, leading to overly general or incomplete summaries compared to the ground truth. SD also had a noticeable overgeneralization rate (4.1%) alongside incorrect value errors (23.6%), reflecting challenges in interpreting ambiguous reports of design elements like blinding or randomisation. Meanwhile, SC and AE were primarily affected by missing field errors (86.5% and 95.5%, respectively), with some factual errors. These results indicate that fields with structured numerical data, such as PC and OM, often have missing or incorrect values, while narrative fields, like IE and SD, are more likely to show overgeneralization errors.

2.6 Error Distribution Across Models with Studies

In order to examine model-specific weakness, we analysed how extraction errors were distributed across combinations of model and studies datasets. Table 2 highlight the five combinations with the highest error

Field	Missing	Incorrect Value	Incorrect Unit	Overgeneralized
Outcome Measures (OM)	95.2% (14,497)	4.3% (660)	0.5% (78)	0.0% (0)
Participant Characteristics (PC)	72.8% (2,522)	24.6% (853)	2.5% (85)	0.2% (6)
Study Design (SD)	71.1% (367)	23.6% (122)	0.0% (0)	5.2% (27)
Study Characteristics (SC)	86.5% (372)	13.5% (58)	0.0% (0)	0.0% (0)
Intervention/Exposure (IE)	64.5% (142)	6.8% (15)	0.0% (0)	28.6% (63)
Adverse Events (AE)	95.5% (461)	3.7% (30)	0.0% (0)	0.2% (1)

Table 1: Top Error type distribution across field groups

counts for each model, showing both the total errors and their breakdown by type. GPT had the highest error counts in most combinations, particularly for OM in studies like MA6 (2,377 errors, 97.5% missing) and MA2 (1,323 errors, 98.5% missing). This shows GPT often misses outcome-related variables. In contrast, Gemini showed a more balanced error distribution, with fewer total errors but a wider range of error types. For instance, in OM for MA6, missing fields accounted for 89.4% of errors, while incorrect values made up 6.1% (79 errors) and incorrect units 2.5% (32 errors), which means Gemini attempts more extraction but struggles with numerical accuracy. Grok also had high missing field rates, such as 94.4% (1,976 errors) in OM for MA6. In OM for MA2, Grok had a higher incorrect value errors (17.7%, 104 errors). Notably, it also produced the relatively fewest errors in the PC field, suggesting stronger performance in handling demographic and baseline variables. Across all models, the OM field presented the greatest challenges, mainly due to high rates of missing information. Analysis of error distribution highlights different models behaviours: GPT takes a cautious approach, often skipping content; Gemini attempts more comprehensive extractions but struggles with numerical precision; and Grok had the fewest errors in PC among the three models.

Model	Field	Studies	Missing	Incorrect Value	Incorrect Unit	Overgeneralized
GPT	OM	MA6	97.5% (2377)	2.3% (56)	0.2% (4)	0.0% (0)
	OM	MA2	98.5% (1323)	1.5% (20)	0.0% (0)	0.0% (0)
	OM	MA5	98.0% (1050)	2.0% (21)	0.0% (0)	0.0% (0)
	OM	MA3	95.9% (674)	4.1% (29)	0.0% (0)	0.0% (0)
	PC	MA6	55.8% (154)	37.0% (102)	7.2% (20)	0.0% (0)
Gemini	OM	MA6	89.4% (1150)	6.1% (79)	2.5% (32)	0.0% (0)
	OM	MA2	96.7% (741)	3.3% (25)	0.0% (0)	0.0% (0)
	OM	MA5	95.7% (633)	2.3% (15)	0.6% (4)	0.0% (0)
	OM	MA3	92.7% (408)	6.8% (30)	0.5% (2)	0.0% (0)
	PC	MA1	72.5% (232)	23.4% (75)	1.3% (4)	0.3% (1)
Grok	OM	MA6	94.4% (1976)	4.8% (101)	0.8% (16)	0.0% (0)
	OM	MA5	91.3% (717)	8.7% (68)	0.0% (0)	0.0% (0)
	OM	MA3	97.4% (589)	2.6% (16)	0.0% (0)	0.0% (0)
	OM	MA2	82.3% (482)	17.7% (104)	0.0% (0)	0.0% (0)
	PC	MA1	55.2% (90)	41.1% (67)	6.1% (10)	1.2% (2)

Table 2: Top 5 error combinations per model across field and task
